# Mechanism of *Synsepalum dulcificum* Daniell. Inhibiting Lung Adenocarcinoma

**DOI:** 10.1155/2022/5242179

**Published:** 2022-02-12

**Authors:** Qi Chen, Tingting Liu, Tuya Bai, Mengdi Zhang, Yuxia Hu, Jun Li, Fuhou Chang

**Affiliations:** ^1^School of Pharmacy, Inner Mongolia Medical University, Hohhot, Inner Mongolia Autonomous Region, China; ^2^New Drug Safety Evaluation Research Center, Inner Mongolia Medical University, Hohhot, Inner Mongolia Autonomous Region, China; ^3^New Drug Screening Engineering Research Center of Inner Mongolia Autonomous Region, Hohhot, Inner Mongolia Autonomous Region, China

## Abstract

*Objective*: *Synsepalum dulcificum* Daniell. (SD) is a natural plant fruit and is famous for containing miraculin. It has been reported that SD can be used as an adjuvant treatment to correct patients' loss of taste during the antitumor process, but the effect of SD itself as an antitumor is not clear. In this study, we investigated the mechanism of action of SD on lung adenocarcinoma using network pharmacology. *Materials and Methods*. The components of SD were identified by liquid chromatography-mass spectrometry, and then the compounds that affect tumor immunity of SD were screened and the related targets were predicted by TCMIO database. At the same time, the results were associated with lung adenocarcinoma targets included in the MalaCards and CTD databases, so as to construct a compound-target action network diagram and explore the mechanism of SD in the treatment of lung adenocarcinoma. In in vitro experiments, cell viability was determined and western blotting was used to detect the related expression of action targets to determine the therapeutic effect of SD. *Results*. In this experiment, 335 chemical components were identified in SD, and 107 components were related to tumor immunity. After screening by ADME, it was found that 11 compounds might be inhaled into the human body and affect the growth of lung adenocarcinoma. In vitro experiments showed that SD could inhibit the growth of lung adenocarcinoma A549 cells. SD could reduce the expression of PCNA (*P* < 0.05) and significantly increase the expression of Caspase-3 (*P* < 0.05). The results of further experiments showed that SD could significantly reduce the phosphorylation of EGFR (*P* < 0.05), and SD could also effectively inhibit the expression of JAK and STAT3 phosphorylation (*P* < 0.01) and inhibit the expression of PI3K and AKT phosphorylation (*P* < 0.01). *Conclusion*. SD can inhibit the growth of lung adenocarcinoma A549 cells and the potential mechanism was found to be the inhibition of EGFR/JAK/STAT3 and EGFR/PI3K/AKT signaling pathway, and the substance basis for SD to exert antitumor effect may be catechin, taxifolin, betaine, epigallocatechin gallate, erucamide, guanosine, kaempferol, lanosterol, morin, oleanolic acid, and quercetin.

## 1. Preface

Lung adenocarcinoma (LUAD) is the most common form of lung cancer, which seriously affects the quality of life of patients and is the cause of death in cancer patients worldwide [1]. Despite tremendous research efforts to develop effective diagnostic techniques and treatments, the overall survival time of LUAD patients is still very short. There is still a lack of treatment options for LUAD patients. Although chemotherapy has made great progress in the treatment of LUAD in recent years, drug resistance is an inevitable outcome, which has a nonnegligible impact on the prognosis and overall survival of patients. Studies have shown that some natural plants can directly inhibit the growth and proliferation of malignant tumors [2]. There are certain functional components of plants, such as alkaloids, dietary polyphenols, and saponins, that are the main functional components of herbs that inhibit the development of lung cancer [3, 4]. However, there is still a lack of natural antitumor plants for clinical use. Therefore, finding new plants that can fight against lung adenocarcinoma and clarifying its pharmacodynamic components is a new method for the treatment of cancer.

*Synsepalum dulcificum* Daniell. (SD) belongs to the mangosteen family [5] and is an evergreen shrub native to tropical West Africa, commonly known as miracle berry, miracle fruit, or miraculous fruit [6]. This is a magical plant with unique features that the sour taste felt by people can be converted into sweet taste [7]. This feature stems from its richness in a sweet glycoprotein called “miracle protein” [8]. All plant parts of SD have medicinal value. Berry fruits and leaves contain many nutrients and many beneficial properties [9, 10]. It has the ability to improve insulin sensitivity, antioxidation, and anticancer ability [11]. Therefore, it can be used as an adjuvant treatment of insulin resistance in diabetic patients [6]. As a valuable plant species, SD is currently used in cosmetics and food. In addition, it is widely used in the pharmaceutical industry [12]. This research mainly focuses on its antitumor properties. At present, there are very limited studies on the types of tumors that this plant can inhibit. Only two compounds found in SD {(+)-syringaresinol and (+)-epi-syringaresinol} have been reported to have inhibitory effects on human skin cancer cells [13]. Studies have also reported the cytotoxic activity of SD berry and stem extracts on colorectal cancer cells (HCT-116, HT-29) and their effects on apoptosis [14]. In the early days of our laboratory, we found that SD has an inhibitory effect on lung adenocarcinoma cells. This study adopts a systematic pharmacological approach to preliminarily explore the mechanism of SD hindering the growth of lung adenocarcinoma, which provides new evidence and basis for the development and utilization of SD, as well as new insights and options for the treatment and prevention of lung cancer.

## 2. Materials and Methods

### 2.1. Identification of SD Components

SD was purchased from Jiangmen, Guangdong Province, China, and was identified as a plant of the genus mysterious fruit of the Solanaceae by a researcher from Inner Mongolia Medical University. The SD samples are stored at −80°C. Methanol, acetonitrile, and formic acid used in this part of the experiment were chromatographic pure, and the other reagents were analytically pure. AB Triple TOF 5600/6600 Mass Spectrometer (AB SCIEX), Agilent 1290 Infinity LC Ultra-High Pressure Liquid Chromatograph (Agilent), and Low Temperature High Speed Centrifuge (Eppendorf 5430R) were used. Column: Waters, ACQUITY UPLC BEH Amide 1.7 *μ*m, 2.1 mm × 100 mm column; Waters, ACQUITY UPLC HSS T3 1.8 *μ*m, 2.1 × 100 mm column.

#### 2.1.1. Sample Pretreatment

First of all, we took an appropriate amount of SD samples, removed the stone, and freeze-dried them into powder for accurate weighing. In order to ensure the stability of the test, the samples were divided into three kinds of samples: peeled pulp (S1), peel (S2), and unpeeled pulp (S3). Preparation of parallel quality control samples (QC): all samples were mixed in equal amounts to prepare QC samples. The QC sample is used to determine the state of the instrument and balance the chromatography-mass spectrometry system before sampling and to evaluate the stability of the system during the entire experiment. Samples were taken out at −80°C, and 80 mg samples were weighed. Then, 200 *μ*l water was added for homogenization, followed by vortexing for 60 s, and 800 *μ*l methanolic acetonitrile solution (1 : 1, v/v) was added, followed by vortexing for 60 s and low-temperature ultrasound for 30 min, twice. Subsequently, the samples were placed at −20°C for 1 h for protein precipitation and centrifuged at 14000 rcf, 4°C for 20 min. Finally, the supernatant was lyophilized, and the samples were stored at −80°C.

#### 2.1.2. Analysis Condition of Liquid Chromatography-Mass Spectrometry

Chromatographic conditions: the sample was separated using Agilent 1290 Infinity LC Ultra-High Performance Liquid Chromatography (UHPLC) HILIC column: column temperature 25b°C; flow rate 0.3 mL/min; mobile phase composition A: water + 25 mM ammonium acetate + 25 mM ammonia, B: acetonitrile; the gradient elution procedure is as follows: 0–0.5 min, 95% B; 0.5–7 min, B linearly changes from 95% to 65%; 7-8 min, B linearly changes from 65% to 40%; 8-9 min, B maintained at 40%; 9-9.1 min, B changed linearly from 40% to 95%; 9.1–12 min, B maintained at 95%; samples were placed in the 4°C autosampler during the entire analysis. In order to ensure the stability of the experiment, the random sequence was adopted for continuous analysis of the samples. QC samples were inserted into the sample queue to monitor and evaluate the stability of the experimental process and the reliability of the experimental data.

2Q-TOF mass spectrometry conditions: Electrospray ionization (ESI) positive ion and negative ion modes are used for detection. The samples were separated by UHPLC and analyzed by Agilent 6550 Mass Spectrometer. The ESI source conditions are as follows: gas temperature: 250°C, drying gas: 16 L/min, nebulizer: 20 psig, sheath gas temperature: 400°C, sheath gas flow: 12 L/min, Vcap: 3000 V, and nozzle voltage: 0 V. Fragment: 175 V, mass range: 50–1200, acquisition rate: 4 Hz, and cycle time: 250 ms. After the sample was tested, the metabolites were identified by AB Triple TOF 6600 Mass Spectrometer, and the primary and secondary spectra of QC samples were collected. The ESI source conditions are as follows: ion source Gas1 (Gas1): 40, ion source Gas2 (Gas2): 80, curtain gas (CUR): 30, source temperature: 650°C, and IonSapary Voltage Floating (ISVF): ±5000 V (positive and negative two modes); the secondary mass spectrum is obtained by information dependent acquisition (IDA), and the high sensitivity mode is adopted, declustering potential (DP): ±60 V (both positive and negative modes), collision energy: 35 ± 15 eV, IDA settings are as follows: exclude isotopes within 4 Da, and candidate ions to monitor per cycle: 10. The data collection is divided into segments according to mass range, 50–300, 290–600, 590–900, and 890–1200, thereby expanding the collection rate of secondary spectra. Each method collects four repetitions per segment. The collected data were used MetDDA and LipDDA methods to identify the structure of metabolites.

### 2.2. Substances of SD Intervention Tumor

In order to find out the substances that affect tumor immunity in SD, we first converted the names of the compounds obtained by liquid-phase technology to the corresponding inchikey identifiers on the PubChem compound website (https://pubchem.ncbi.nlm.nih.gov/) for subsequent analysis. After that, we used the TCMIO database (http://tcmio.xielab.net/) to screen compounds related to tumor immunity in SD. Through the analysis of the results of the previous pharmacokinetic studies on the active ingredients of natural plants, it is very necessary to further adopt the ADME evaluation of the ingredients. In this study, the parameters of SD potential absorption components were set as human oral bioavailability (OB) ≥ 20% and drug similarity (DL) ≥ 0.18 [15]. Finally, we showed more detailed ADME data for the screened compounds using the SwissADME (http://www.swissadme.ch/) online tool.

### 2.3. Target of Predictive Screening Component

In this part, we used the method of predicting the corresponding targets of small-molecule compounds in the SwissTargetPrediction tool (http://www.swisstargetprediction.ch/) to collect the screened targets of compounds and analyzed the species to select human in the prediction process and excluded the data with the credible value of 0. When the SwissTargetPrediction tool failed to query the target data of certain compounds, we used the target data included in the TCMSP (https://www.tcmsp-e.com/) database to supplement this blank.

### 2.4. Targets of Lung Adenocarcinoma

For targeted information on lung adenocarcinoma, we chose to obtain it from the MalaCards database (https://www.malacards.org/). We searched the disease module of the database for genetic information about lung adenocarcinoma and screened elite genes, which means that the selection results are highly correlated with lung adenocarcinoma. In order to ensure the comprehensiveness of the data, we also merged the lung adenocarcinoma-related gene information collected in the CTD database (http://ctdbase.org/). In this database, only the genes that are directly related to the disease were selected. After integrating the relevant information on the diseases, we crossed them with the targets that SD affects and thus obtained the targets that SD may affect lung adenocarcinoma.

### 2.5. Protein-Protein Interaction

In order to fully understand the pharmacological mechanism of SD on lung adenocarcinoma, we performed protein-protein interaction (PPI) analysis on the target. The target names obtained in the previous step are input into the string (https://www.string-db.org/) website, and the research species are selected human. The protein interaction selection was only obtained from the experimental conditions, and no more than 20 interaction objects were selected in the expanded two-layer interaction numbers. The confidence selection was greater than 0.7, and the rest settings were set as the system default. We imported the interaction information into Cytoscape software to draw the protein-protein interaction network, used Generate Style tool to use the node size and color to reflect the degree value and the edge thickness setting to reflect the comprehensive score, and finally obtained PPI [16].

### 2.6. Bio-Functional Enrichment

In this part, we used the results obtained in 2.5 to perform routine biological function enrichment so that we can understand the functions and positioning of these proteins and other information. Function enrichment mainly used David online tools (https://david.ncifcrf.gov/). Enrichment items included biological process (BP), location in the cell (CC), and molecular function (MF) under the gene ontology (GO). Kyoto Encyclopedia of Genes and Genomes (KEGG) in pathway analysis was selected as pathway analysis. The analysis process conditions were set as significance *P* < 0.01, and the auxiliary screening conditions FDR < 0.01.

### 2.7. Bioinformatics Analysis of Targets

Differential expression and clinical significance of lung adenocarcinoma targets affected by SD are the primary basis for ensuring SD to have a clear therapeutic effect. We analyzed the details of the target gene in lung adenocarcinoma using the GEPIA 2.0 (http://gepia2.cancer-pku.cn/#index) database. First, we verified the expression differences of the genes obtained in Section 2.4 between the lung adenocarcinoma and the control group. The target gene was input into the expression module, and the analysis conditions were set as | Log 2FC |; the cutoff value was the default value of 1, and the cutoff value of *P* value was the default value of 0.01. The tumor type was LUAD, and log 2 (TPM + 1) was used as the logarithmic scale. Then, among the targets with clear differences, we continued to search for genes related to prognosis. The genes were input into the survival analysis column of the website, and only the information of overall survival was collected. Group cutoff chose the median method, and cutoff-high (%) and cutoff-low (%) both chose 50. The risk ratio was calculated according to Cox PH model. Target expression determines whether a patient can be successfully distinguished from a control group, which is one of the important bases for targeting therapeutic targets. To achieve this, principal component analysis (PCA) was performed on genes with clear differences.

### 2.8. Immune Infiltration Analysis of Targets

In screening the active ingredients of SD against lung adenocarcinoma, all compounds selected from the TCMIO database were related to tumor immunity, so it was necessary to carry out immune infiltration analysis against the target. In this part of the work, data were collected using Timer 2.0 (https://cistrome.shinyapps.io/timer/) database. The differential genes obtained in the previous part were mainly input to the immune module. The types of immune cells were selected as B cells, CD8+T cells, CD4+T cells, macrophages, neutrophils, and dendritic cells.

### 2.9. In Vitro Experiment Part

#### 2.9.1. Cell Culture

Human lung adenocarcinoma cell line A549 was cultured in DMEM containing 10% fetal bovine serum and 1% antibiotics in a cell incubator at 37°C and 5% CO_2_.

#### 2.9.2. Effects of SD on Cytotoxicity

The cells cultured in advance were counted by using a blood cell counting plate. The cell suspension was diluted by a certain factor and the cell density was adjusted to 5000 cells/mL and added into a 96-well plate, which was divided into 10 drug concentration gradients, 1, 2, 4, 8, 16, 32, 64, 128, 256, and 512 *μ*g/ml. Five wells were set for each drug concentration, as well as a control well and a zero adjustment well. After placed in a 37°C cell incubator for 24 h, drugs were added. After stimulation with drugs for 20 h, 20 *μ*L of MTT solution was added into each well for incubation at 37°C for 4 h, and then 150 *μ*L of dimethyl sulfoxide (DMSO) was added into each well for shaking for 10 min to fully dissolve the formazan. The average proliferation rate of cells in each group of wells was calculated and the IC50 curve was plotted using log-logistic method (effect minimum value 0 and effect maximum value 1). The preparation method of the medicine was that the SD freeze-dried powder was dissolved by DMSO. In our subsequent mechanistic studies, the dose concentration of 1/2 time the IC50, 1 time the IC50, and 2 times the IC50 was selected as the low, medium, and high dose.

#### 2.9.3. Western Blot Analysis

The cultured cells were taken for immunoblot analysis. After lysis and centrifugation, the sample supernatant was quantified by BCA protein analysis kit. Then, protein samples were separated by 10% polyacrylamide gel electrophoresis. Protein was transferred to polyvinylidene fluoride membrane and blocked with 5% skim milk to prevent nonspecific binding and then incubated overnight with appropriate antibodies and primary antibodies of internal reference at 4°C. After culture, the cell membrane was washed with TBST and then cultured with appropriate secondary antibody labeled with peroxidase. Compared with *β*-actin expression, all protein blots expressed average area density. Antibodies were sourced from Abcam, specific product information is anti-EGFR (ab52894), anti-EGFR (phosphoY1068, ab40815), anti-JAK1 (ab133666), anti-JAK1 (phosphoY1022 + Y1023, ab13805), anti-STAT3 (ab68153), anti-STAT3 (phosphoY705, ab76315), anti-PI3K (ab191606), anti-PI3K (phosphoY607, ab182651), anti-AKT1 (ab179463), and anti-AKT1 (phosphoS473, ab81283).

### 2.10. Statistical Analysis

The experimental data were analyzed using SPSS 22.0 statistical software. All experiments were repeated at least three times. Data are presented as mean standard deviation. One-way analysis of variance was used to compare the means of multiple groups of independent samples, and the result when *P* < 0.05 was considered to be statistically significant.

## 3. Results

### 3.1. Components of SD

**Figure 1:**
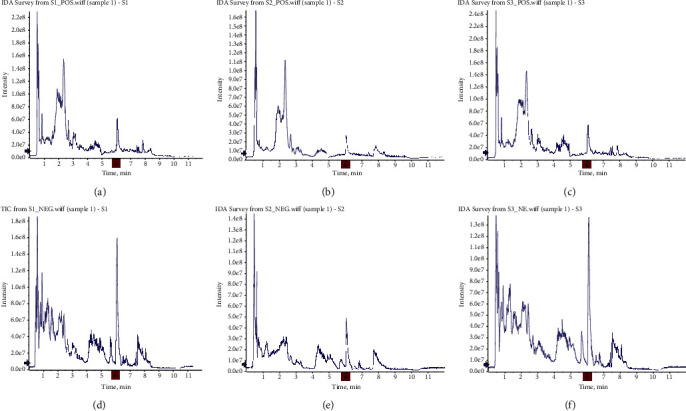
TIC diagram of SD sample (anion and cation diagrams of S1, S2, and S3 samples, respectively).

The total ion chromatogram (TIC) obtained from the analysis of samples by UHPLC-Q-TOF MS is shown in Figure 1. Through the retention time (tR), fragmentation pattern, and sample search in the database, the results of the three samples were combined and deduplicated to obtain a total of 355 components of SD. The component information is shown in Table 1.

### 3.2. Compounds of SD Intervention Tumor

After search comparison, we found 107 relevant components of SD that could interfere with tumor immunity in the TCMIO database, and the compound details are populated in Table 2. Eleven active components were obtained after screening by ADME, and the molecular weight and OB and DL parameters of these eleven compounds are shown in Table 3. The ADME values for these compounds are presented in Supplementary Table 1.

### 3.3. The Relationship between Compound and Lung Adenocarcinoma Targets

**Figure 2:**
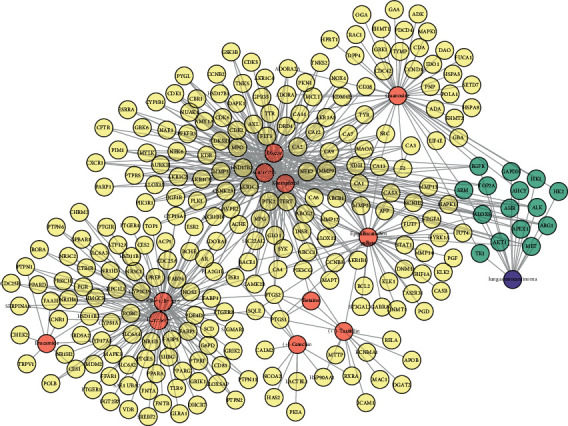
Compound-action target-disease-network topology (red figure represents SD compound with potential absorption capacity, yellow graphic represents the predicted target of the compound, blue represents disease and associated action targets, and the C-T-D topology has a total of 270 nodes and 582 edges).

After summarizing the results, we obtained 255 related targets for 11 compounds. There were 23 targets of lung adenocarcinoma in MalaCards database and 159 targets in CTD database, and a total of 177 targets related to lung cancer were obtained after eliminating duplicate values. After the intersection with the targets affected by SD was completed, a total of 15 action targets were obtained. This indicates SD can affect lung adenocarcinoma through multiple targets. By using Cytoscape software to construct the network topology diagram of “chemical composition-potential action target-lung adenocarcinoma target” (Figure 2), we can more intuitively display the action target of SD. The C-T-D topology is constructed with a total of 270 nodes and 582 edges.

### 3.4. Result of PPI and Functional Enrichment

**Figure 3:**
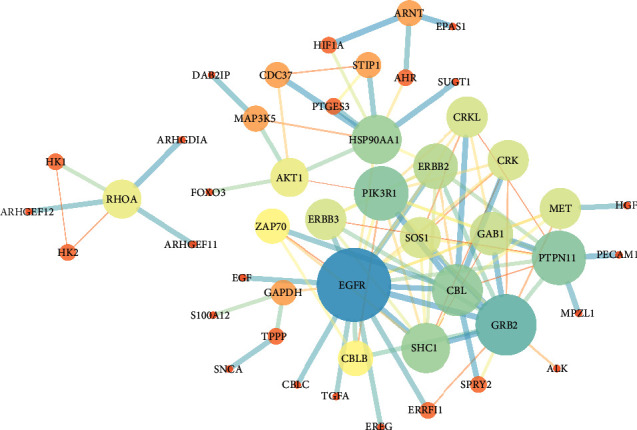
PPI network diagram of SD acting on lung adenocarcinoma targets (48 targets and 101 edges are shown in the figure, where the points with relatively large area represent hub genes, and the thickness of connecting line represents combined score).

There were 48 targets and 101 edges in PPI network diagram (Figure 3). Among them, large spots represented key genes. The biological processes of target enrichment included ERBB2 signal pathway, epidermal growth factor receptor signal pathway, and phosphatidylinositol-mediated signal transduction. The target genes were mainly located in the cytoplasm, membrane raft, plasma membrane, etc. The molecular functions performed involve protein tyrosine kinase activity, protein phosphatase binding, epidermal growth factor receptor binding, guanylate exchange factor activity, etc. The enriched signal pathways included ErbB signal pathway, cancer pathway, proteoglycan in cancer, and neurotrophic factor signal pathway. More detailed enrichment data results are stored in Table 4. The above results reflect that lung adenocarcinoma, as a complex disease, involves many biological processes, and SD can play a therapeutic role by regulating these biological processes. Through the analysis in this part, it is found that SD in the intervention of lung adenocarcinoma not only acts on a single target but as a herb containing rich compounds that plays an anticancer role with multiple targets. Previous studies have shown that natural plants acting on multiple targets always play a good role in inhibiting lung adenocarcinoma [17, 18].

### 3.5. Bioinformatics Results

**Figure 4:**
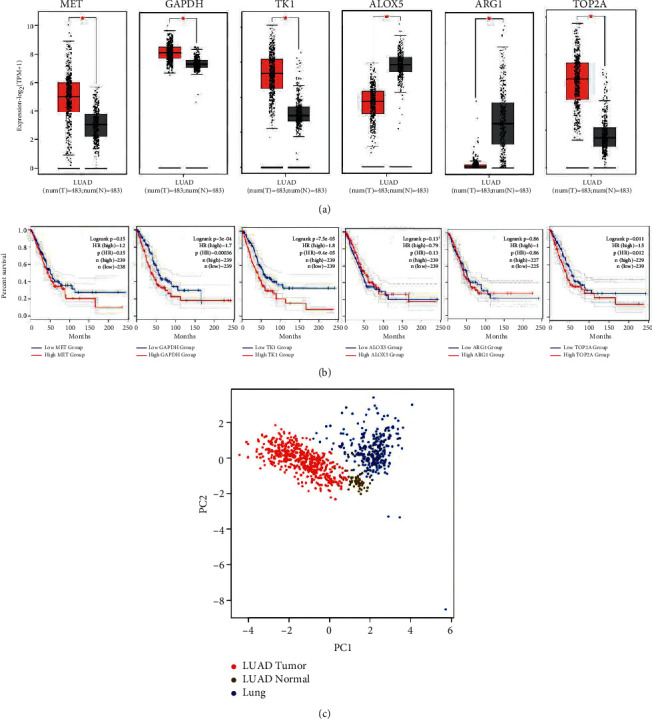
Expression of targets where SD affects lung adenocarcinoma and survival prognosis information. (a) Six genes with clear differences. (b) Six genes survival information in lung adenocarcinoma. (c) Six genes PCA dimension reduction diagram in lung adenocarcinoma and control group. ^*∗*^*P* < 0.05.

The results indicated that there were 6 target genes with different expression in lung adenocarcinoma, namely, MET proto-oncogene (MET), glyceraldehyde-3-phosphate dehydrogenase (GAPDH), thymidine kinase 1 (TK1), arachidonate 5-lipoxygenase (ALOX5), arginase 1 (ARG1), and DNA topoisomerase II alpha (TOP2A) (Figure 4(a)). Compared with their respective control groups, the expressions of MET, GAPDH, TK1, and TOP2A increased (*P* < 0.01), while the expressions of ALOX5 and ARG1 decreased (*P* < 0.01). Among the survival information results, the prognosis of GAPDH with high expression was worse and statistically different from that of low expression group (*P* < 0.01), while the prognosis of high expression group of TK1 was worse (*P* < 0.01), and the prognosis of high expression group of TOP2A was poor (*P* < 0.05) (Figure 4(b)). Six genes with clear differential expression were included in principal component analysis, and PCA results showed that the selection of target gene could distinguish the expression of lung adenocarcinoma from that of the control group (Figure 4(c)). Studies have found that the progression of lung adenocarcinoma is not always characterized by mutations in a single gene or protein, but when patient diagnosed with lung adenocarcinoma, it is always accompanied by changes in multiple genes, RNA, or enzymes [19]. This indicates that the target drugs designed for the characteristics of lung adenocarcinoma should target multiple targets to play a role, which is also the advantage of natural plants as drugs for the treatment of lung adenocarcinoma.

### 3.6. Results of Immune Infiltration of Genes

**Figure 5:**
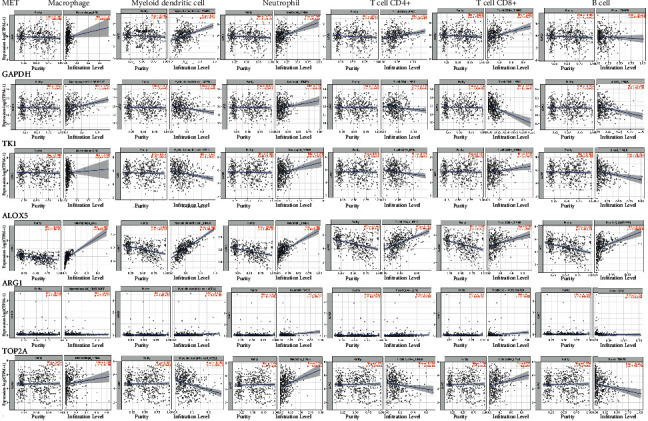
Relationship between six target genes and immune scores (correlation of MET, GAPDH, TK1, ALOX5, ARG1, and TOP2A genes with immune infiltration scores of B cells, CD8+T cells, CD4+T cells, macrophages, neutrophils, and dendritic cells).

In recent decades, more and more attention has been paid to tumor microenvironment, which includes the infiltration of immune cells. We found that patients with LUAD with high risk score had a higher proportion of activated CD4+T cells, NK cells, M0 and M1 macrophages, and activated mast cells [20]. The six hub genes selected here are all highly correlated with B cells, CD8+T cells, CD4+T cells, macrophages, neutrophils, and dendritic cells (Figure 5), which offers potential applications for cancer immunotherapy. Remarkably, tumor-infiltrating immune cells in lung cancer may be an important determinant of prognosis and immunotherapy response [21, 22]. However, further experiments are needed to explore new biomarkers and the complex mechanisms of immune cells.

### 3.7. Results of In Vitro Experiments

#### 3.7.1. Cytotoxicity Results of SD

**Figure 6:**
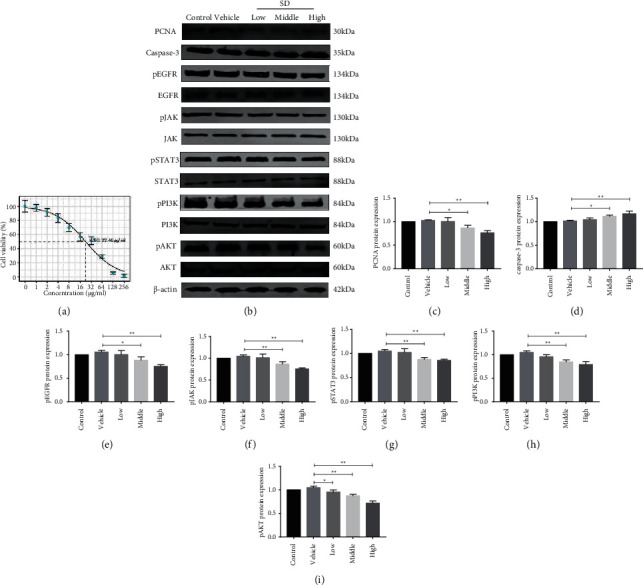
Effect of SD on A549 cytotoxicity and the expression of related factors. (a) The inhibitory effect of SD on cell growth. (b) The effect of SD on A549 cell proliferation and apoptosis and EGFR signaling pathway-related proteins. (c–i) Protein band statistical diagram. ^*∗∗*^*P* < 0.01, ^*∗*^*P* < 0.05.

According to the value-added rate of MTT detection, it was found that compared with the blank group, the cell viability was decreased with the increase of drug concentration. When the concentration of SD was >8 *μ*g/ml, the cell viability was significantly decreased (*P* < 0.01). The IC50 of SD for this preparation was calculated as 22.49 *μ*g/mL (Figure 6(a)). The concentrations in the low, medium, and high dose groups for subsequent experiments were selected as 10 *μ*g/ml, 20 *μ*g/ml, and 40 *μ*g/ml.

#### 3.7.2. Effects of SD on A549 Proliferation and Apoptosis

The results of proliferative cell nuclear antigen (PCNA), a classical marker of cell proliferation and apoptosis, and Caspase-3 were used to explore the effect of SD on A549 cell growth rate. PCNA is an acidic nucleic acid protein and a polypeptide necessary for DNA synthesis that specifically reflects the cell proliferation state. It is an endogenous nuclear protein specifically expressed in cells at the proliferative phase. Caspase-3 is a key signal regulatory protein that promotes cancer cell apoptosis in Caspase family. The expression of both proteins can rapidly and accurately evaluate tumor proliferation and apoptosis capacity at the molecular biological level, which has important guiding value for the selection of clinical treatment plan and prognosis judgment. In order to further study the mechanism of SD affecting the growth of tumor cells, in the network pharmacology part, we have observed that the epidermal growth factor receptor signaling pathway is an important medium for SD affecting lung adenocarcinoma, and in the in vitro part, we have verified the mechanism of SD affecting the epidermal growth factor receptor-related pathways, focusing on EGFR/JAK/STAT and EGFR/PI3K/AKT.

Specifically, compared with the blank group, the proteins of the solvent control group were not significantly changed (*P* > 0.05). Compared with the solvent control group, the expression of PCNA was significantly decreased in the medium and high dose drug treatment groups (*P* < 0.05), and the expression of Caspase-3 was significantly increased in the medium and high dose drug treatment groups (*P* < 0.05). Moreover, compared with the control group, the phosphorylation of EGFR was significantly reduced in the medium and high dose treatment groups (*P* < 0.05), and the drug at this concentration could effectively inhibit the phosphorylation expression of JAK and STAT3 (*P* < 0.01). Finally, compared with the solvent control group, the medium and high dose medication group significantly inhibited the expression of PI3K phosphorylation (*P* < 0.01), while the low, medium, and high dose medication group significantly inhibited the expression of AKT phosphorylation (*P* < 0.01).

## 4. Discussion

Although lung cancer has remained as a type of cancer with a high mortality rate over the past decades, there have been significant improvements in the way lung cancer is diagnosed and treated [23]. In recent years, targeted therapeutic drugs such as epidermal growth factor receptor tyrosine kinase inhibitor (EGFR-TKIs) and anaplastic lymphoma kinase (ALK) inhibitor, as well as immunotherapy drugs targeting programmed cell death protein 1 or programmed death ligand 1, have greatly improved the therapeutic effect of lung cancer and provided additional treatment options for patients with advanced and refractory lung cancer [24]. Although EGFR-TKI is very effective in the treatment of EGFR mutant lung cancer, resistance to these agents develops within an average of about 1 year [25]. Therefore, overcoming the drug resistance of EGFR-targeted related drugs remains one of the difficulties in the development of antitumor drugs. Natural plants are rich in a variety of compounds, which can well avoid the resistance of chemical drugs to a single target, which is also the main reason of traditional Chinese medicine as one of the most important complementary and alternative medicine types.

Like many advantages of natural plants, SD also affects many targets in the process of inhibiting tumor. Based on the literature, we describe the relatively important targets. MET is a tyrosine kinase receptor encoding hepatocyte growth factor, which can promote the proliferation, migration, and invasion of tumor cells by binding HGF ligand. Recent studies have found that about 3%∼5% of NSCLC patients have MET mutation, and 1%∼5% of them show MET amplification [26]. At the same time, it is found that MET gene amplification is independently related to CD8+T cell infiltration level, and the infiltration rate of CD8+T cells in NSCLC is as high as 68.9%, which indicates that MET may become a potential target for immunotherapy of NSCLC [27]. GAPDH plays a role in glycolysis and nuclear transcription, RNA transport, DNA replication, and apoptosis. Studies have pointed out that the expression of GAPDH in lung cancer, kidney cancer, breast cancer, and other tumors is out of control [28]. It has been found that the high expression of GAPDH is related to the proliferation and invasion of lung cancer and esophageal cancer [29]. In addition, research based on RNA-binding protein-related prognosis model has pointed out that the prognosis model containing GAPDH can better diagnose and predict the survival time of LUAD patients [30]. TK1 is a cytoplasmic enzyme involved in pyrimidine metabolism that catalyzes the addition of *γ*-phosphate groups to thymidine. TK1 has been studied as a biomarker for the diagnosis and prognosis of many types of cancer, including lung cancer [31]. In addition, the missing TK1 has been shown to inhibit the growth and metastatic ability of lung adenocarcinoma in vitro and in mice by reducing the expression of growth differentiation factors [32]. ALOX5 is a member of the family of genes encoding lipoxygenase and plays a dual role in the synthesis of leukotrienes from arachidonic acid. Mutations in the promoter region of this gene may be associated with several cancers. ALOX5 is considered to be a candidate biomarker for noninvasive molecular diagnosis of lung cancer [33]. ARG1 is a cytoplasmic enzyme that is mainly expressed in the liver. At the same time, as a part of urea cycle, it is also expressed in immune cells of peripheral blood. ARG1 metabolizes L-arginine into urea and L-ornithine and generates proline and polyamines downstream, which is crucial for cell proliferation and collagen synthesis [34]. Previous studies have revealed the fact that ARG1 is involved in anti-inflammation, tumor immunity, and immunosuppression-related diseases. The results have demonstrated that ARG1 may play a key role in the progression of hepatocellular carcinoma by promoting the EMT process. Systemic or bone marrow-specific ARG1 deletions can improve antigen-induced proliferation of adoptive transferred T cells and lead to inhibition of lung cancer tumor growth. These results suggest that ARG1, as an oncogene, may play a role in lung adenocarcinoma [35]. TOP2A is an enzyme that can change and control the DNA topological state during transcription. Abnormal expression of this enzyme has been shown to be associated with increased risk of tumor metastasis, drug resistance, and abnormal cell cycle [36, 37]. At the same time, it is considered to be the target of several anticancer drugs, such as etoposide and topotecan [38]. In addition, TOP2A is upregulated in various tumors such as colon and ovarian malignant tumors and can be used as a sensitive biomarker for early detection and treatment of these tumors [39]. In summary, we have found that the targets affected by SD play a very important role in lung cancer, which indicates that SD synergistically exerts the antilung cancer effect through multiple impact targets.

To clarify the material basis of SD acting on multiple targets, we constructed a network topology diagram of SD acting on lung adenocarcinoma and analyzed the main components that may play a role as catechin, taxifolin, betaine, epigallocatechin gallate (EGCG), erucamide, guanosine, kaempferol, lanosterol, morin, oleanolic acid, and quercetin. Previous numerous studies have shown that these substances have antitumor effects. For example, EGCG is the most abundant and bioactive catechin. Studies have shown that it can inhibit tumor proliferation and metastasis and induce lung cancer cell apoptosis in vitro and in vivo [40]. In addition, EGCG can also inhibit the growth of lung cancer cells through Ras-GTPase activating protein SH3 domain binding protein-1 [41]. At the same time, as an inhibitor of neutrophil elastase, EGCG could block the migration of A549 cells induced by neutrophil elastase by up-regulating AAT expression [42]. Furthermore, guanosine also has excellent antitumor activity and significant inhibitory activity on lung adenocarcinoma A5449 cell line [43]. Kaempferol and quercetin are the most widely studied antitumor substances; it has been reported that Kaempferol can reduce the expression of CLDN2 in A549 cells and further inhibit cell proliferation and migration [44]. It has been demonstrated that the anticancer activity of quercetin is facilitated via the activation of the adenosine monophosphate (AMP)-activated protein kinase (AMPK) pathway, suppression of the phosphoinositide 3-kinase/PI3K/AKT/mammalian target of rapamycin/NF-*κ*B pathway, upregulation of p53 activation, and the apoptosis pathway, and when used with other chemotherapeutic agents, quercetin can achieve tumor-improving results by enhancing apoptosis and reducing side effects [45]. Based on the research results of this experiment and literature reports, we believe that SD has the effect of multiple natural compounds affecting multiple targets to play an antilung adenocarcinoma role.

At the same time, in the part of network pharmacology, we found that an important signaling pathway for the intervention of SD on lung adenocarcinoma was EGFR signaling pathway. In the experimental part, we focused on the mechanism of SD affecting the proliferation and apoptosis of A549 cells through EGFR. EGFR is a receptor tyrosine kinase that transduces a signal cascade across that plasma membrane (from the extracellular environment to the intracellular environment). EGFR is highly expressed in a variety of malignant tumors, and its receptor dimerization can activate JAK1 and STAT, thereby regulating the cell cycle and apoptosis of lung cancer cells [46]. The continuous activation of STAT3 is closely related to malignant transformation of cells and participates in the occurrence and development of various tumors. Studies have found that constitutive activation of STAT3 can be detected in lung cancer, so it is considered to be closely related to the occurrence and development of lung cancer. Subsequent studies have further revealed abnormal activation of STAT3 in approximately 55% of NSCLC patients and in most NSCLC cell lines. This activation is more common in patients with small tumors, patients with a short history of smoking, and patients with lung adenocarcinoma [47]. In addition, the phosphorylation of EGFR activates phosphatidylinositol 3-kinase (PI3K), which activates downstream signaling molecules in the pathway and promotes the proliferation, infiltration, and metastasis of tumor cells [48]. Thus, effective control of the expression of JAK/STAT3 and PI3K/AKT by EGFR contributes to the regulation of the growth and apoptotic state of tumor cells. We have found in the experiment that SD can effectively inhibit the growth of lung adenocarcinoma cells from the point of view of the effect of SD on the proliferation of A549 cells, and we have also found that SD can inhibit the expression of PCNA and increase the expression of Caspase-3. We further found that SD significantly inhibited the phosphorylated expression of EGFR, which in turn inhibited the phosphorylated expression of JAK/STAT and PI3K/AKT. This indicated that SD had the effect of inhibiting the growth of lung adenocarcinoma cells, and the mechanism might be related to the inhibition of EGFR pathway.

## 5. Conclusion

In the whole experiment, we used different technical means to identify the medicinal value of SD as a natural plant against lung adenocarcinoma. First of all, we identified the chemical constituents of SD by liquid chromatography-mass spectrometry and related the compound information with the medicinal materials database and then mined out the key compounds in SD that interfered with tumor. It is found that the main substances that exert the efficacy of SD are catechin, taxifolin, betaine, epigallocatechin gallate, erucamide, guanosine, kaempferol, lanosterol, morin, oleanolic acid, and quercetin. SD containing the main components acts through different targets, among which the more important targets are MET, GAPDH, TK1, ALOX5, ARG1, and TOP2A. We also found that SD affected various signaling pathways to play a pharmacological role against the growth of lung adenocarcinoma cells, including ERBB2 signaling pathway, epidermal growth factor receptor signaling pathway, and phosphatidylinositol-mediated signaling. Finally, the in vitro experiments showed that SD could block the EGFR/JAK/STAT and EGFR/PI3K/AKT signaling pathways to different degrees to inhibit the proliferation of lung adenocarcinoma A549 cells and increase apoptosis. In summary, SD has a significant anticancer effect on lung adenocarcinoma A549 cells. Next, whether SD exerts the same antitumor effect in vivo is the direction of our main experiment.

## Figures and Tables

**Table 1:** Information on the identification of compounds by liquid phase-mass spectrometry.

Name	m/z	rt (s)	Adduct	Description	Inchikey
M787T108	786.5989258	107.95	(M+H)+	1,2-Dioleoyl-sn-glycero-3-phosphatidylcholine	SNKAWJBJQDLSFF-NVKMUCNASA-N
M127T286	127.0374405	286.359	(M+H)+	1,3,5-Benzenetriol	QCDYQQDYXPDABM-UHFFFAOYSA-N
M295T37_2	295.2242494	37.285	(M+Na)+	16-Hydroxy hexadecanoic acid	QOHPSSZLXKNRIP-UHFFFAOYSA-N
M102T282	102.0542526	282.309	(M+H)+	1-Aminocyclopropanecarboxylic acid	PAJPWUMXBYXFCZ-UHFFFAOYSA-N
M193T203	193.0848282	203.474	(M+CH_3_COO+2H)+	1-Indanone	IHMQOBPGHZFGLC-UHFFFAOYSA-N
M522T182_2	522.3543806	181.995	(M+H)+	1-Oleoyl-sn-glycero-3-phosphocholine	YAMUFBLWGFFICM-PTGWMXDISA-N
M454T198_2	454.2930393	198.334	(M+H)+	1-Palmitoyl-2-hydroxy-sn-glycero-3-phosphoethanolamine	YVYMBNSKXOXSKW-HXUWFJFHSA-N
M313T150	313.2723127	150.278	(M+H−H_2_O)+	1-Palmitoylglycerol	QHZLMUACJMDIAE-UHFFFAOYSA-N
M496T191_2	496.339664	191.335	(M+H)+	1-Palmitoyl-sn-glycero-3-phosphocholine	ASWBNKHCZGQVJV-HSZRJFAPSA-N
M524T187_2	524.3698134	187.365	(M+H)+	1-Stearoyl-2-hydroxy-sn-glycero-3-phosphocholine	IHNKQIMGVNPMTC-UHFFFAOYSA-N
M482T194	482.3221438	193.995	(M+H)+	1-Stearoyl-2-hydroxy-sn-glycero-3-phosphoethanolamine	BBYWOYAFBUOUFP-JOCHJYFZSA-N
M341T160	341.3030714	160.087	(M+H−H_2_O)+	1-Stearoyl-rac-glycerol	VBICKXHEKHSIBG-UHFFFAOYSA-N
M70T310_2	70.06513209	309.757	(M+H−2H_2_O)+	2-Amino-2-methyl-1,3-propanediol	UXFQFBNBSPQBJW-UHFFFAOYSA-N
M151T8	151.0949437	8.418	(M+CH_3_COO+2H)+	2-Ethoxyethanol	XZDUGACICAWSKQ-UHFFFAOYSA-N
M152T198	152.0558511	197.634	(M+H)+	2-Hydroxyadenine	DRAVOWXCEBXPTN-UHFFFAOYSA-N
M298T197	298.1130365	196.884	(M+H)+	2-Methylguanosine	SLEHROROQDYRAW-KQYNXXCUSA-N
M325T415	325.11161	415.121	(M+H−H_2_O)+	3.Alpha.-mannobiose	QIGJYVCQYDKYDW-UHFFFAOYSA-N
M102T68_2	102.0535141	67.663	(M+NH_4_)+	3-Butynoic acid	LWNHDEQKHFRYMD-UHFFFAOYSA-N
M146T374	146.116097	373.753	M+	(3-Carboxypropyl)trimethylammonium cation	
M300T45	300.2886281	44.744	(M+H)+	3-Ketosphinganine	KBUNOSOGGAARKZ-KRWDZBQOSA-N
M138T184	138.0540965	184.035	(M+H)+	4-Aminobenzoate	ALYNCZNDIQEVRV-UHFFFAOYSA-M
M104T285	104.0695112	285.049	(M+H)+	4-Aminobutyric acid	BTCSSZJGUNDROE-UHFFFAOYSA-N
M146T356	146.0911306	356.165	(M+H)+	4-Guanidinobutyric acid	TUHVEAJXIMEOSA-UHFFFAOYSA-N
M87T407	87.04320855	406.551	(M+H)+	4-Hydroxybutanoic acid lactone	YEJRWHAVMIAJKC-UHFFFAOYSA-N
M147T297	147.042935	297.358	(M+H−H_2_O)+	4-Hydroxycinnamic acid	NGSWKAQJJWESNS-ZZXKWVIFSA-N
M184T46	184.0596548	46.174	(M+H)+	4-Pyridoxic acid	HXACOUQIXZGNBF-UHFFFAOYSA-N
M170T247_2	170.0802161	247.001	(M+H)+	6-Hydroxydopamine	DIVDFFZHCJEHGG-UHFFFAOYSA-N
M282T96	282.1697336	96.361	(M+NH_4_)+	Abscisic acid (cis, trans)	JLIDBLDQVAYHNE-QHFMCZIYSA-N
M810T429	810.1311067	428.76	(M+H)+	Acetyl coenzyme A (Acetyl-CoA)	ZSLZBFCDCINBPY-ZSJPKINUSA-N
M136T167	136.0610335	166.906	(M+H)+	Adenine	GFFGJBXGBJISGV-UHFFFAOYSA-N
M268T170	268.1045728	170.386	(M+H)+	Adenosine	OIRDTQYFTABQOQ-KQYNXXCUSA-N
M330T361	330.0580254	361.274	(M+H)+	Adenosine 2′,3′-cyclic monophosphate	KMYWVDDIPVNLME-UHFFFAOYSA-N
M243T474	243.0251281	474.287	(M+H−H_2_O)+	alpha-D-Glucose 1-phosphate	HXXFSFRBOHSIMQ-VFUOTHLCSA-N
M296T36	296.2566095	35.955	(M+NH_4_)+	alpha-Linolenic acid	DTOSIQBPPRVQHS-PDBXOOCHSA-N
M138T357	138.0541369	356.894	(M+H)+	Anthranilic acid (Vitamin L1)	RWZYAGGXGHYGMB-UHFFFAOYSA-N
M245T229	245.1473991	229.032	M+	Arg-Ala	WVRUNFYJIHNFKD-WDSKDSINSA-N
M350T182	350.2050038	181.995	(M+CH_3_CN+H)+	Bestatin	XGDFITZJGKUSDK-UDYGKFQRSA-N
M130T106	130.0846404	106.02	(M+H)+	.beta.-Homoproline	ADSALMJPJUKESW-RXMQYKEDSA-N
M118T556	118.0847563	555.7875	(M+H)+	Betaine	KWIUHFFTVRNATP-UHFFFAOYSA-N
M335T463	335.0624921	462.787	M+	beta-Nicotinamide D-ribonucleotide	DAYLJWODMCOQEW-TURQNECASA-N
M583T234	583.2525148	233.772	(M+H)+	Biliverdin	RCNSAJSGRJSBKK-NSQVQWHSSA-N
M291T42	291.0849661	41.954	(M+H)+	(+)-Catechin	PFTAWBLQPZVEMU-DZGCQCFKSA-N
M104T353	104.1056977	353.254	M+	Choline	OEYIOHPDSNJKLS-UHFFFAOYSA-N
M192T374	192.0866954	374.423	(M+CH_3_COO+2H)+	cis-4-Hydroxy-D-proline	PMMYEEVYMWASQN-QWWZWVQMSA-N
M175T493	175.0228574	492.956	(M+H)+	cis-Aconitate	GTZCVFVGUGFEME-IWQZZHSRSA-N
M210T478	210.059606	477.617	(M+NH_4_)+	Citrate	KRKNYBCHXYNGOX-UHFFFAOYSA-K
M449T261	449.1045143	260.54	M+	Cyanidin 3-glucoside cation	RKWHWFONKJEUEF-GQUPQBGVSA-O
M160T380_2	160.133592	380.373	(M+CH_3_COO+2H)+	Cyclohexylamine	PAFZNILMFXTMIY-UHFFFAOYSA-N
M244T239	244.0910047	238.752	(M+H)+	Cytidine	UHDGCWIWMRVCDJ-XVFCMESISA-N
M404T480	404.0201169	479.586	(M+H)+	Cytidine 5′-diphosphate (CDP)	ZWIADYZPOWUWEW-XVFCMESISA-N
M112T252_2	112.0491632	252.261	(M+H)+	Cytosine	OPTASPLRGRRNAP-UHFFFAOYSA-N
M161T109	161.090403	109.22	(M+H)+	D-Alanyl-D-alanine (D-Ala-D-Ala)	BYXHQQCXAJARLQ-HSUXUTPPSA-N
M134T261	134.0438816	260.54	(M+H)+	D-Aspartic acid	CKLJMWTZIZZHCS-UWTATZPHSA-N
M323T500	322.9913052	500.465	(M+H−H_2_O)+	D-Fructose 1,6-bisphosphate	RNBGYGVWRKECFJ-ZXXMMSQZSA-N
M261T459	261.0351507	459.0025	(M+H)+	D-Glucose 6-phosphate	VFRROHXSMXFLSN-SLPGGIOYSA-N
M609T201	609.1795129	200.514	(M+H)+	Diosmin	GZSOSUNBTXMUFQ-YFAPSIMESA-N
M162T372	162.0752579	371.764	(M+H)+	DL-2-Aminoadipic acid	OYIFNHCXNCRBQI-UHFFFAOYSA-N
M188T256	188.0714014	255.721	(M+H−H_2_O)+	DL-Indole-3-lactic acid	XGILAAMKEQUXLS-UHFFFAOYSA-N
M198T259	198.096718	259.121	(M+NH_4_)+	D-Mannose	WQZGKKKJIJFFOK-QTVWNMPRSA-N
M136T216	136.0773049	215.793	(M+H−H_2_O)+	Dopamine	VYFYYTLLBUKUHU-UHFFFAOYSA-N
M130T539	130.0859092	539.054	(M+H)+	D-Pipecolinic acid	HXEACLLIILLPRG-RXMQYKEDSA-N
M116T358	116.0698598	358.344	(M+H)+	D-Proline	ONIBWKKTOPOVIA-SCSAIBSYSA-N
M459T176	459.0895832	175.616	(M+H)+	Epigallocatechin gallate	WMBWREPUVVBILR-WIYYLYMNSA-N
M145T414	145.0484017	414.431	(M+Na)+	Erythritol	UNXHWFMMPAWVPI-ZXZARUISSA-N
M786T400	786.1631491	400.471	(M+H)+	Flavin adenine dinucleotide (FAD)	VWWQXMAJTJZDQX-UYBVJOGSSA-N
M308T493	308.088006	492.956	(M+H)+	Glutathione	RWSXRVCMGQZWBV-WDSKDSINSA-N
M613T509	613.1550342	508.945	(M+H)+	Glutathione disulfide	YPZRWBKMTBYPTK-BJDJZHNGSA-N
M258T349	258.1160825	348.935	M+	Glycerophosphocholine	SUHOQUVVVLNYQR-MRVPVSSYSA-N
M284T261	284.0985394	261.221	(M+H)+	Guanosine	NYHBQMYGNKIUIF-UUOKFMHZSA-N
M285T394	285.1188498	394.072	(M+H)+	His-Glu	VHOLZZKNEBBHTH-YUMQZZPRSA-N
M269T295	269.1599267	294.778	(M+H)+	His-Ile	IDXZDKMBEXLFMB-UHFFFAOYSA-N
M132T435	132.0643176	434.71	(M+H)+	Hydroxyproline	PMMYEEVYMWASQN-DMTCNVIQSA-N
M465T264	465.0986109	264	(M+H)+	Hyperoside	OVSQVDMCBVZWGM-DTGCRPNFSA-N
M513T151	513.2627198	150.957	(2M+Na)+	Ile-Asn	HZYHBDVRCBDJJV-UHFFFAOYSA-N
M261T292	261.1428887	291.558	(M+H)+	Ile-Glu	KTGFOCFYOZQVRJ-ZKWXMUAHSA-N
M189T265	189.1221063	265.32	(M+H)+	Ile-Gly	UCGDDTHMMVWVMV-FSPLSTOPSA-N
M245T103	245.1918811	102.681	(M+H)+	Ile-Ile	BCVIOZZGJNOEQS-XKNYDFJKSA-N
M263T189	263.1417649	188.675	(M+H)+	Ile-Met	TUYOFUHICRWDGA-CIUDSAMLSA-N
M229T247	229.1532714	247.001	(M+H)+	Ile-Pro	BBIXOODYWPFNDT-CIUDSAMLSA-N
M118T256_2	118.0639514	255.721	(M+H)+	Indole	SIKJAQJRHWYJAI-UHFFFAOYSA-N
M221T295	221.0900328	295.418	(M+NH_4_)+	Indole-3-pyruvic acid	RSTKLPZEZYGQPY-UHFFFAOYSA-N
M170T256	170.0585649	255.721	(M+H−H_2_O)+	Indoleacrylic acid	SWYBZVHYBMBHJS-UHFFFAOYSA-N
M409T33_2	409.3808315	32.625	(M+H−H_2_O)+	Lanosterol	CAHGCLMLTWQZNJ-BQNIITSRSA-N
M174T248_2	174.1120928	248.491	M+	L-Arginine	ODKSFYDXXFIFQN-BYPYZUCNSA-N
M133T444	133.0595501	444.4585	(M+H)+	L-Asparagine	DCXYFEDJOCDNAF-REOHCLBHSA-N
M134T464	134.0435111	464.208	(M+H)+	L-Aspartate	CKLJMWTZIZZHCS-REOHCLBHSA-N
M162T393	162.1105883	393.362	(M+H)+	L-Carnitine	PHIQHXFUZVPYII-ZCFIWIBFSA-N
M203T96	203.1406329	96.361	(M+H)+	Leu-Ala	HSQGMTRYSIHDAC-BQBZGAKWSA-N
M260T286	260.1595396	286.359	(M+H)+	Leu-Gln	JYOAXOMPIXKMKK-YUMQZZPRSA-N
M245T185	245.185141	185.325	(M+H)+	Leu-Leu	LCPYQJIKPJDLLB-UWVGGRQHSA-N
M260T263	260.1949277	263.31	(M+H)+	Leu-Lys	OTXBNHIUIHNGAO-UWVGGRQHSA-N
M279T173	279.1691237	172.966	(M+H)+	Leu-Phe	KFKWRHQBZQICHA-STQMWFEESA-N
M219T268	219.1325735	267.92	(M+H)+	Leu-Ser	XGDCYUQSFDQISZ-BQBZGAKWSA-N
M233T56	233.1488733	55.943	(M+H)+	Leu-Thr	LRKCBIUDWAXNEG-CSMHCCOUSA-N
M318T184	318.1801535	184.035	(M+H)+	Leu-Trp	BQVUABVGYYSDCJ-ZFWWWQNUSA-N
M231T198	231.1702781	198.334	(M+H)+	Leu-Val	MDSUKZSLOATHMH-IUCAKERBSA-N
M148T459	148.0592457	459.278	(M+H)+	L-Glutamate	WHUUTDBJXJRKMK-VKHMYHEASA-N
M156T397	156.0756208	396.872	(M+H)+	L-Histidine	HNDVDQJCIGZPNO-YFKPBYRVSA-N
M118T50	118.029661	49.824	(M+H−H_2_O)+	L-Homocysteine	FFFHZYDWPBMWHY-VKHMYHEASA-N
M298T76	298.2716847	76.072	(M+NH_4_)+	Linoleic acid	OYHQOLUKZRVURQ-HZJYTTRNSA-N
M132T261	132.1010124	261.221	(M+H)+	L-Leucine	ROHFNLRQFUQHCH-YFKPBYRVSA-N
M150T285	150.056382	285.049	(M+H)+	L-Methionine	FFEARJCKVFRZRR-BYPYZUCNSA-N
M166T277	166.0831806	277.349	(M+H)+	L-Phenylalanine	COLNVLDHVKWLRT-QMMMGPOBSA-N
M130T295	130.0846047	294.778	(M+H)+	L-Pipecolic acid	HXEACLLIILLPRG-YFKPBYRVSA-N
M147T286	147.0752091	285.699	(M+NH_4_)+	L-Pyroglutamic acid	ODHCTXKNWHHXJC-VKHMYHEASA-N
M259T456	259.1278766	455.868	(M+H−H_2_O)+	L-Saccharopine	ZDGJAHTZVHVLOT-YUMQZZPRSA-N
M120T408	120.0641743	407.851	(M+H)+	L-Threonine	AYFVYJQAPQTCCC-GBXIJSLDSA-N
M351T189	351.1311706	189.375	(M+CH_3_CN+Na)+	Lycorine	ZOERACVSGIQXBP-CANOEZFNSA-N
M307T264	307.1667248	264	(M+CH_3_CN+Na)+	Lys-Pro	AIXUQKMMBQJZCU-IUCAKERBSA-N
M846T506	846.3031535	506.415	(M+NH_4_)+	Maltopentaose	BADXJDLPQWRZFL-NZYQVXNASA-N
M522T427	522.2006691	426.51	(M+NH_4_)+	Maltotriose	HNKASWRMLBJLKJ-HNNWOXMSSA-N
M221T48	221.0944979	47.654	(M+H)+	Met-Ala	JHKXZYLNVJRAAJ-WDSKDSINSA-N
M277T407_2	277.1488765	406.551	M+	Met-Lys	IMTUWVJPCQPJEE-IUCAKERBSA-N
M337T190	337.2718782	190.025	(M+H−H_2_O)+	MG (18 : 2(9Z,12Z)/0 : 0/0 : 0)[rac]	WECGLUPZRHILCT-HZJYTTRNSA-N
M319T203_2	319.0439066	202.734	(M+H)+	Myricetin	IKMDFBPHZNJCSN-UHFFFAOYSA-N
M189T385	189.1224279	385.052	(M+H)+	N6-Acetyl-L-lysine	DTERQYGMUDWYAZ-ZETCQYMHSA-N
M282T291	282.1169803	290.908	(M+H)+	N6-Methyladenosine	VQAYFKKCNSOZKM-IOSLPCCCSA-N
M189T562	189.1598982	562.3865	(M+H)+	N6,N6,N6-Trimethyl-L-lysine	MXNRLFUSFKVQSK-QMMMGPOBSA-N
M188T401	188.1748166	401.221	(M+H)+	N8-Acetylspermidine	FONIWJIDLJEJTL-UHFFFAOYSA-N
M203T543	203.1490301	542.6335	(M+H)+	NG,NG-Dimethyl-L-arginine (ADMA)	SYLNVYJOPZWPJI-ILKKLZGPSA-N
M123T64	123.0544031	64.073	(M+H)+	Nicotinamide	DFPAKSUCGFBDDF-UHFFFAOYSA-N
M744T492	744.0772818	492.226	(M+H)+	Nicotinamide adenine dinucleotide phosphate (NADP)	XJLXINKUBYWONI-NNYOXOHSSA-L
M124T385	124.0376678	385.052	(M+H)+	Nicotinate	PVNIIMVLHYAWGP-UHFFFAOYSA-M
M110T55	110.0593247	55.193	(M+H)+	Nicotinyl	MVQVNTPHUGQQHK-UHFFFAOYSA-N
M247T35	247.241756	34.645	(M+H−2H_2_O)+	Oleic acid	ZQPPMHVWECSIRJ-OLLJCFGNSA-N
M279T68_2	279.136174	67.663	(M+H)+	Pantetheine	ZNXZGRMVNNHPCA-VIFPVBQESA-N
M220T277_3	220.1175406	276.659	(M+H)+	Pantothenate	GQTHJBOWLPZUOI-FJXQXJEOSA-M
M757T121	756.5539728	120.689	(M+Na)+	PC (16 : 0/16 : 0)	KILNVBDSWZSGLL-KXQOOQHDSA-N
M237T297	237.128132	296.718	(M+H)+	Phe-Ala	MIDZLCFIAINOQN-WPRPVWTQSA-N
M294T263	294.1428086	262.61	(M+H)+	Phe-Gln	KLAONOISLHWJEE-QWRGUYRKSA-N
M303T181	303.1387042	181.326	(M+H)+	Phe-His	OHUXOEXBXPZKPT-STQMWFEESA-N
M137T292	137.0582761	291.558	(M+H)+	Phenylacetic acid	WLJVXDMOQOGPHL-UHFFFAOYSA-N
M313T88	313.159347	87.751	(M+H)+	Phe-Phe	GKZIWHRNKRBEOH-HOTGVXAUSA-N
M267T213	267.1322735	212.993	(M+H)+	Phe-Thr	NYQBYASWHVRESG-MIMYLULJSA-N
M352T189_2	352.1710986	188.675	(M+H)+	Phe-Trp	JMCOUWKXLXDERB-WMZOPIPTSA-N
M184T545	184.0720253	544.917	(M+H)+	Phosphorylcholine	YHHSONZFOIEMCP-UHFFFAOYSA-N
M318T149	318.2998121	148.978	(M+H)+	Phytosphingosine	AERBNCYCJBRYDG-KSZLIROESA-N
M187T359	187.1068128	359.044	(M+H)+	Pro-Ala	FELJDCNGZFDUNR-WDSKDSINSA-N
M579T269	579.1452665	269.24	(M+H)+	Procyanidin B2	XFZJEEAOWLFHDH-NFJBMHMQSA-N
M245T386	245.1124492	386.413	(M+H)+	Pro-Glu	QLROSWPKSBORFJ-BQBZGAKWSA-N
M150T94	150.0534145	94.301	(M+H−H_2_O)+	Pyridoxal (Vitamin B6)	NGVDGCNFYWLIFO-UHFFFAOYSA-N
M170T152	170.0805554	152.317	(M+H)+	Pyridoxine	LXNHXLLTXMVWPM-UHFFFAOYSA-N
M303T179	303.0488019	179.131	(M+H)+	Quercetin	REFJWTPEDVJJIY-UHFFFAOYSA-N
M317T157	317.0638016	156.587	(M+H)+	Quercetin 3′-methyl ether	WEPBGSIAWZTEJR-UHFFFAOYSA-N
M449T157	449.1060127	156.587	(M+H)+	Quercitrin	OXGUCUVFOIWWQJ-HQBVPOQASA-N
M193T268	193.0687226	267.92	(M+H)+	Quinate	AAWZDTNXLSGCEK-LNVDRNJUSA-N
M522T465	522.2004096	464.897	(M+NH_4_)+	Raffinose	MUPFEKGTMRGPLJ-ZQSKZDJDSA-N
M298T99	298.096902	99.211	(M+H)+	S-Methyl-5′-thioadenosine	WUUGFSXJNOTRMR-IOSLPCCCSA-N
M216T393	216.0626181	392.642	(M+H)+	sn-Glycerol 3-phosphoethanolamine	SLKDGVPOSSLUAI-PGUFJCEWSA-N
M302T41	302.3031789	40.585	(M+H)+	Sphinganine	OTKJDMGTUTTYMP-ZWKOTPCHSA-N
M684T510	684.2540314	510.205	(M+NH_4_)+	Stachyose	UQZIYBXSHAGNOE-XNSRJBNMSA-N
M360T638	360.1477882	638.124	(M+NH_4_)+	Sucrose	CZMRCDWAGMRECN-UGDNZRGBSA-N
M305T218	305.0650308	218.423	(M+H)+	(+-)-Taxifolin	CXQWRCVTCMQVQX-LSDHHAIUSA-N
M265T247	265.1135071	247.001	M+	Thiamine	JZRWCGZRTZMZEH-UHFFFAOYSA-N
M759T182	758.5642603	181.995	(M+Na)+	Thioetheramide-PC	GUTOVRMLPRGBNS-UHFFFAOYSA-N
M235T423	235.0913282	423.46	(M+H)+	Thr-Asp	IOWJRKAVLALBQB-IWGUZYHVSA-N
M225T128	225.0747524	127.789	(M+CH_3_COO+2H)+	trans-3-Coumaric acid	KKSDGJDHHZEWEP-SNAWJCMRSA-N
M114T345	114.0533519	344.975	(M+H−H_2_O)+	trans-4-Hydroxy-L-proline	PMMYEEVYMWASQN-DMTCNVIQSA-N
M195T128	195.0640504	128.439	(M+H)+	trans-Ferulic acid	KSEBMYQBYZTDHS-HWKANZROSA-N
M265T35	265.2506952	34.645	(M+H−H_2_O)+	trans-Vaccenic acid	UWHZIFQPPBDJPM-BQYQJAHWSA-N
M213T417	213.121983	417.17	(M+CH_3_CN+Na)+	Triethanolamine	GSEJCLTVZPLZKY-UHFFFAOYSA-N
M253T252	253.1169612	251.531	(M+H)+	Tyr-Ala	NLKUJNGEGZDXGO-XVKPBYJWSA-N
M138T244	138.0901305	243.911	(M+H)+	Tyramine	DZGWFCGJZKJUFP-UHFFFAOYSA-N
M283T279	283.1226401	278.739	(M+H)+	Tyr-Thr	MFEVVAXTBZELLL-UHFFFAOYSA-N
M584T449	584.0867724	448.918	(M+NH_4_)+	UDP-D-Galactose	HSCJRCZFDFQWRP-LNYDKVEPSA-N
M425T33_2	425.3731394	32.625	(M+H−H_2_O)+	Uvaol	XUARCIYIVXVTAE-ZAPOICBTSA-N
M189T96_2	189.1231719	95.651	(M+H)+	Val-Ala	HSRXSKHRSXRCFC-WDSKDSINSA-N
M247T370	247.1279565	369.783	(M+H)+	Val-Glu	UPJONISHZRADBH-XPUUQOCRSA-N
M265T272	265.1555874	271.98	(M+H)+	Val-Phe	GJNDXQBALKCYSZ-RYUDHWBXSA-N
M260T76	260.1589293	76.072	(M+CH_3_CN+H)+	Val-Thr	GVRKWABULJAONN-VQVTYTSYSA-N
M165T148	165.0184514	148.378	(M−H)−	1,2-Benzenedicarboxylic acid	XNGIFLGASWRNHJ-UHFFFAOYSA-N
M671T178	671.462351	177.646	(M−H)−	1-Palmitoyl-2-linoleoyl-sn-glycero-3-phosphate	YQMUIZXKIKXZHD-UMKNCJEQSA-N
M748T37	747.5155779	37.235	(M−H)−	1-Palmitoyl-2-oleoyl-phosphatidylglycerol	PAZGBAOHGQRCBP-DDDNOICHSA-N
M153T249	153.0183535	249.082	(M−H)−	2,3-Dihydroxybenzoic acid	GLDQAMYCGOIJDV-UHFFFAOYSA-N
M243T126	243.0245474	126.099	(M−H)−	2-Deoxy-D-glucose 6-phosphate	UQJFZAAGZAYVKZ-CERMHHMHSA-N
M193T182	193.0715163	182.446	(M+CH_3_COO)−	2′-Deoxy-D-ribose	ASJSAQIRZKANQN-CRCLSJGQSA-N
M273T155	273.0374996	155.228	(M+CH_3_COO)−	2-Deoxyribose 5-phosphate	KKZFLSZAWCYPOC-VPENINKCSA-N
M141T398	141.0178968	397.782	(M−H_2_O−H)−	2-Oxoadipic acid	FGSBNBBHOZHUBO-UHFFFAOYSA-N
M171T249	171.0290613	249.082	(M−H)−	3-Dehydroshikimic acid	SLWWJZMPHJJOPH-PHDIDXHHSA-N
M225T295	225.0876832	294.749	(M+K−2H)−	3-Hydroxycapric acid	FYSSBMZUBSBFJL-UHFFFAOYSA-N
M373T227	373.1847516	226.923	(M+K−2H)−	5(S)-HpETE	JNUUNUQHXIOFDA-JGKLHWIESA-N
M263T95	263.1279804	95.471	(M−H)−	(+)-Abscisic acid	JLIDBLDQVAYHNE-YKALOCIXSA-N
M426T462	426.020217	462.218	(M−H)−	Adenosine 5′-diphosphate (ADP)	XTWYTFMLZFPYCI-KQYNXXCUSA-N
M558T426	558.0611793	426.21	(M−H)−	ADP-ribose	SRNWOUGRCWSEMX-KEOHHSTQSA-N
M515T158	515.2975562	158.038	(M−H)−	Adynerin	BYZQVAOKDQTHHP-QFUJVLJYSA-N
M277T45	277.2153721	45.075	(M−H)−	All cis-(6,9,12)-Linolenic acid	VZCCETWTMQHEPK-QNEBEIHSSA-N
M179T257	179.0562544	256.881	(M−H)−	alpha-D-Glucose	WQZGKKKJIJFFOK-DVKNGEFBSA-N
M331T158	331.1020108	158.038	(M+CH_3_COO)−	Arbutin	BJRNKVDFDLYUGJ-RMPHRYRLSA-N
M144T23	144.044448	23.436	(M+NH_4_−2H)−	Barbituric acid	HNYOPLTXPVRDBG-UHFFFAOYSA-N
M401T250_1	401.1387353	249.812	(M+CH_3_COO)−	Cellobiose	GUBGYTABKSRVRQ-QRZGKKJRSA-N
M465T27	465.3029207	26.806	(M−H)−	Cholesteryl sulfate	BHYOQNUELFTYRT-DPAQBDIFSA-N
M253T46	253.2154284	45.735	(M−H)−	cis-9-Palmitoleic acid	SECPZKHBENQXJG-FPLPWBNLSA-N
M129T455	129.0189355	455.148	(M−H)−	Citraconic acid	HNEGQIOMVPPMNR-IHWYPQMZSA-N
M359T304	359.1174188	303.588	(2M−H)−	D-Allose	BZVNQJMWJJOFFB-FGTMMUONSA-N
M207T73	207.0502776	73.023	(M+CH_3_COO)−	D-Arabinono-1,4-lactone	MYRODPRKOYUJTI-JJYYJPOSSA-N
M487T273	487.1759595	273.32	(2M−H)−	D-Biotin	XGTGBTVIPQNPMG-ROCTVOAFSA-N
M347T60	347.0241224	60.184	(2M−H)−	Dehydroascorbic acid (Oxidized vitamin C)	SBJKKFFYIZUCET-JLAZNSOCSA-N
M359T259_2	359.1170934	259.401	(2M−H)−	D-Fructose	LKDRXBCSQODPBY-VRPWFDPXSA-N
M191T286	191.0187151	285.599	(M−H_2_O−H)−	D-Galactarate	DSLZVSRJTYRBFB-DUHBMQHGSA-N
M193T392	193.0341903	391.853	(M−H)−	D-Galacturonic acid	AEMOLEFTQBMNLQ-YMDCURPLSA-N
M195T443	195.0498835	442.9195	(M−H)−	D-gluconate	RGHNJXZEOKUKBD-SQOUGZDYSA-M
M237T248	237.0603697	247.702	(M+CH_3_COO)−	D-Glucono-1,5-lactone	PHOQVHQSTUBQQK-SQOUGZDYSA-N
M175T249	175.0238724	249.082	(M−H)−	D-Glucuronolactone	UYUXSRADSPPKRZ-SKNVOMKLSA-N
M113T377	113.0358489	377.003	(M−H)−	Dihydrouracil	OIVLITBTBDPEFK-UHFFFAOYSA-N
M71T258	71.01381752	258.151	(M−H_2_O−H)−	Dihydroxyacetone	RXKJFZQQPQGTFL-UHFFFAOYSA-N
M209T215	209.0656764	215.159	(M+CH_3_COO)−	D-Lyxose	SRBFZHDQGSBBOR-AGQMPKSLSA-N
M223T514	223.0790504	513.885	(M+CH_3_COO)−	D-Quinovose	SHZGCJCMOBCMKK-GASJEMHNSA-N
M149T157	149.0444752	156.617	(M−H)−	D-Ribose	SRBFZHDQGSBBOR-SOOFDHNKSA-N
M159T434	159.0101313	433.59	(M+K−2H)−	D-Threitol	UNXHWFMMPAWVPI-QWWZWVQMSA-N
M221T353	221.0655515	352.835	(M−H)−	Ethyl glucuronide	IWJBVMJWSPZNJH-UQGZVRACSA-N
M401T412	401.127448	411.741	(M+CH_3_COO)−	Galactinol	VCWMRQDBPZKXKG-ZNVDUFQESA-N
M195T379	195.0503617	378.953	(M−H)−	Galactonic acid	RGHNJXZEOKUKBD-MGCNEYSASA-N
M165T429	165.0399132	428.88	(M+CH_3_COO)−	Glyceric acid	RBNPOMFGQQGHHO-UHFFFAOYSA-N
M187T376	187.0234439	376.374	(M−H_2_O−H)−	Homocitrate	XKJVEVRQMLKSMO-SSDOTTSWSA-N
M167T124	167.034665	124.15	(M−H)−	Homogentisic acid	IGMNYECMUMZDDF-UHFFFAOYSA-N
M364T507	364.0528857	506.845	(M−H)−	Indapamide	NDDAHWYSQHTHNT-UHFFFAOYSA-N
M383T453	383.0441996	453.319	(2M−H)−	Isocitrate	ODBLHEXUDAPZAU-UHFFFAOYSA-N
M163T224	163.0608555	224.053	(M−H)−	L-Fucose	SHZGCJCMOBCMKK-DHVFOXMCSA-N
M145T374	145.0606406	373.834	(M−H)−	L-Glutamine	ZDXPYRJPNDTMRX-VKHMYHEASA-N
M177T140	177.0394182	140.409	(M−H)−	L-Gulonic gamma-lactone	SXZYCXMUPBBULW-SKNVOMKLSA-N
M409T244	409.1689548	243.652	M−	Linustatin	FERSMFQBWVBKQK-CXTTVELOSA-N
M133T405	133.0148751	404.972	(M−H)−	L-Malic acid	BJEPYKJPYRNKOW-REOHCLBHSA-N
M163T251	163.0585946	251.141	(M−H)−	L-Rhamnose	SHZGCJCMOBCMKK-JFNONXLTSA-N
M161T182	161.0451717	182.446	(M−H_2_O−H)−	L-Sorbose	LKDRXBCSQODPBY-AMVSKUEXSA-N
M135T355	135.0298096	354.765	(M−H)−	L-Threonate	JPIJQSOTBSSVTP-STHAYSLISA-M
M114T377	114.0189933	377.003	(M−H)−	Maleamic acid	FSQQTNAZHBEJLS-UPHRSURJSA-N
M325T335	325.1098389	334.546	(M−H_2_O−H)−	Maltitol	VQHSOMBJVWLPSR-WUJBLJFYSA-N
M471T52	471.3414816	52.344	(M−H)−	Maslinic Acid	MDZKJHQSJHYOHJ-LLICELPBSA-N
M213T178	213.0163458	178.336	M−	m-Chlorohippuric acid	ICYUIIJXZHPESK-UHFFFAOYSA-N
M117T128	117.0188608	127.7095	(M−H)−	Methylmalonic acid	ZIYVHBGGAOATLY-UHFFFAOYSA-N
M207T223	207.0863285	222.643	(M+CH_3_COO)−	Mevalonic acid	KJTLQQUUPVSXIM-ZCFIWIBFSA-N
M179T394	179.0556848	394.442	(M−H)−	myo-Inositol	CDAISMWEOUEBRE-UHFFFAOYSA-N
M211T282	211.071326	281.72	(M+CH_3_COO)−	N1-Methyl-2-pyridone-5-carboxamide	JLQSXXWTCJPCBC-UHFFFAOYSA-N
M301T44	301.0576135	43.755	M−	N-Acetyl-D-Glucosamine 6-Phosphate	BRGMHAYQAZFZDJ-RTRLPJTCSA-N
M442T352	442.1528631	351.575	(M+CH_3_COO)−	N-Acetyl-D-lactosamine	KFEUJDWYNGMDBV-RPHKZZMBSA-N
M190T291	190.0508256	290.789	(M−H)−	N-Acetyl-DL-methionine	XUYPXLNMDZIRQH-UHFFFAOYSA-N
M164T68	164.0350457	67.533	(M−H)−	N-Formylanthranilic acid	LLLPDUXGHXIXIW-UHFFFAOYSA-N
M823T137	823.4487299	137.109	(M−H)−	Nodularin	IXBQSRWSVIBXNC-HSKGSTCASA-N
M233T68	233.0661615	67.533	(M+Na−2H)−	Perseitol	OXQKEKGBFMQTML-RYRJNEICSA-N
M435T144	435.1272196	143.698	(M−H)−	Phloridzin	IOUVKUPGCMBWBT-QNDFHXLGSA-N
M865T257	865.1962754	256.881	(M−H)−	Procyanidin C1	MOJZMWJRUKIQGL-XILRTYJMSA-N
M190T374	190.070972	374.464	(M+CH_3_COO)−	Propionylglycine	WOMAZEJKVZLLFE-UHFFFAOYSA-N
M143T147	143.034251	147.028	(2M−H)−	Pyruvaldehyde	AIJULSRZWUXGPQ-UHFFFAOYSA-N
M383T357	383.1182533	357.355	(2M−H)−	Quinic acid	AAWZDTNXLSGCEK-LNVDRNJUSA-N
M133T250	133.049834	250.452	(M−H_2_O−H)−	Ribitol	HEBKCHPVOIAQTA-NGQZWQHPSA-N
M147T457	147.0294963	456.978	(M−H)−	(S)-2-Hydroxyglutarate	HWXBTNAVRSUOJR-VKHMYHEASA-N
M137T293	137.0235166	293.459	(M−H)−	Salicylic acid	YGSDEFSMJLZEOE-UHFFFAOYSA-N
M173T186	173.0437859	185.746	(M−H)−	Shikimate	JXOHGGNKMLTUBP-HSUXUTPPSA-N
M283T9	283.2624045	8.921	(M−H)−	Stearic acid	QIQXTHQIDYTFRH-UHFFFAOYSA-N
M117T388	117.019444	387.943	(M−H)−	Succinate	KDYFGRWQOYBRFD-UHFFFAOYSA-L
M343T88	343.0679782	88.292	(M−H)−	Thiamine monophosphate	HZSAJDVWZRBGIF-UHFFFAOYSA-O
M606T437	606.073559	436.96	(M−H)−	UDP-N-Acetylglucosamine	LFTYTUAZOPRMMI-CFRASDGPSA-N
M565T448	565.0463944	448.239	(M−H)−	Uridine diphosphate glucose (UDP-D-glucose)	HSCJRCZFDFQWRP-JZMIEXBBSA-N
M276T458	276.1539123	458.063	(M+H)+	.gamma.-L-Glu-.epsilon.-L-Lys	JPKNLFVGUZRHOB-SFYZADRCSA-N
M550T178	550.3821504	178.051	M+	1-O-(cis-9-Octadecenyl)-2-O-acetyl-sn-glycero-3-phosphocholine	ZBOQHUSCQCEBGK-UHFFFAOYSA-O
M295T68_2	295.2249385	67.988	(M+Na)+	16-Hydroxypalmitic acid	UGAGPNKCDRTDHP-UHFFFAOYSA-N
M195T129	195.0637798	128.914	(M+H)+	3-Hydroxy-4-methoxycinnamic acid	QURCVMIEKCOAJU-HWKANZROSA-N
M259T492	259.0200137	491.701	(M+H−H_2_O)+	6-Phospho-D-gluconate	OVPRPPOVAXRCED-WVZVXSGGSA-N
M217T340	217.1463693	339.92	M+	Ala-Lys	QXRNAOYBCYVZCD-BQBZGAKWSA-N
M209T297	209.1161676	296.663	(M+CH_3_COO+2H)+	Anethole	NEPQOSMFDZLYOO-UHFFFAOYSA-N
M449T208	449.1044771	207.899	(M+H)+	Astragalin	JPUKWEQWGBDDQB-QSOFNFLRSA-N
M278T459	278.0617148	458.753	(M+NH_4_)+	D-Mannose-6-phosphate	OBHLNVXMRZXIII-MVNLRXSJSA-M
M70T393	70.06432841	393.287	(M+H−2H_2_O)+	Diethanolamine	ZBCBWPMODOFKDW-UHFFFAOYSA-N
M330T74	330.2617644	73.837	M+	Eicosapentaenoic Acid ethyl ester	SSQPWTVBQMWLSZ-AAQCHOMXSA-N
M338T34_2	338.3405989	34.34	(M+H)+	Erucamide	UAUDZVJPLUQNMU-KTKRTIGZSA-N
M227T428	227.0626666	427.945	(M+Na)+	Gly-Glu	IEFJWDNGDZAYNZ-BYPYZUCNSA-N
M247T367	247.1273668	367.039	(M+H)+	Ile-Asp	WKXVAXOSIPTXEC-UHFFFAOYSA-N
M274T257	274.1759142	256.816	(M+CH_3_CN+H)+	Ile-Thr	DRCKHKZYDLJYFQ-UHFFFAOYSA-N
M85T73	85.06402465	73.087	(M+H−H_2_O)+	Isovaleric acid	GWYFCOCPABKNJV-UHFFFAOYSA-N
M378T304	378.1584614	304.053	(2M+NH_4_)+	L-(-)Sorbose	BEZJAQLKYOEUBD-ARFHVFGLSA-N
M102T49	102.0538519	49.009	(M+H)+	L-.alpha.-Amino-.gamma.-butyrolactone	QJPWUUJVYOJNMH-GSVOUGTGSA-N
M129T225	129.100622	225.148	(M +H−H_2_O)+	L-Lysine	KDXKERNSBIXSRK-YFKPBYRVSA-N
M288T348	288.200496	347.67	(M+H)+	Leu-Arg	SENJXOPIZNYLHU-IUCAKERBSA-N
M243T62	243.0869941	62.088	(M+H)+	Lumichrome	ZJTJUVIJVLLGSP-UHFFFAOYSA-N
M262T454	262.1410424	453.538	(M+H)+	Lys-Asp	CIOWSLJGLSUOME-BQBZGAKWSA-N
M297T177	297.1246426	176.731	(M+H)+	Met-Phe	HGCNKOLVKRAVHD-RYUDHWBXSA-N
M249T396	249.1425708	396.157	(M+CH_3_CN+H)+	Miglitol	IBAQFPQHRJAVAV-ULAWRXDQSA-N
M206T73	206.1375505	73.087	M+	Monoethylglycinexylidide (MEGX)	WRMRXPASUROZGT-UHFFFAOYSA-N
M176T459	176.0900628	459.463	(M+H)+	N-Carboxyethyl-.gamma.-aminobutyric acid	SRGQUICKDUQCKO-UHFFFAOYSA-N
M175T363	175.1069418	363.139	(M+H)+	N2-Acetyl-L-ornithine	JRLGPAXAGHMNOL-LURJTMIESA-N
M265T221	265.1622388	220.888	M+	Oxprenolol	CEMAWMOMDPGJMB-UHFFFAOYSA-N
M295T347	295.1278752	346.95	(M+H)+	Phe-Glu	JXWLMUIXUXLIJR-QWRGUYRKSA-N
M253T238	253.117338	237.677	(M+H)+	Phe-Ser	ROHDXJUFQVRDAV-UWVGGRQHSA-N
M215T205	215.13818	205.039	(M+H)+	Pro-Val	AWJGUZSYVIVZGP-UHFFFAOYSA-N
M399T551	399.1432851	550.987	(M+H)+	S-Adenosylmethionine	MEFKEPWMEQBLKI-AIRLBKTGSA-N
M235T420	235.0918264	420.055	(M+H)+	Ser-Glu	LAFKUZYWNCHOHT-UHFFFAOYSA-N
M502T320	502.2065795	320.412	(2M+NH_4_)+	Thymidine	UBTJZUKVKGZHAD-UPRLRBBYSA-N
M318T184_2	318.1798249	183.58	(M+H)+	Trp-Ile	PITVQFJBUFDJDD-XEGUGMAKSA-N
M295T207	295.1634377	207.189	(M+H)+	Tyr-Ile	QJKMCQRFHJRIPU-XDTLVQLUSA-N
M231T199	231.1694195	199.219	(M+H)+	Val-Ile	PNVLWFYAPWAQMU-CIUDSAMLSA-N
M246T34	246.1414554	33.68	(M+CH_3_CN+H)+	Val-Ser	STTYIMSDIYISRG-UHFFFAOYSA-N
M237T369	237.0601184	368.639	(M+CH_3_COO)−	2-Dehydro-3-deoxy-D-gluconate	OVPRPPOVAXRCED-WVZVXSGGSA-N
M759T74	758.5667436	74.468	(M−H)−	2-Oleoyl-1-palmitoyl-sn-glycero-3-phosphocholine (PC (16 : 0/18 : 1(9Z)))	WTJKGGKOPKCXLL-RYDYYDTQSA-N
M161T397	161.0444239	396.508	(M−H)−	3-Hydorxy-3-methylglutaric acid	NPOAOTPXWNWTSH-UHFFFAOYSA-N
M99T378	99.04487504	377.539	(M−H_2_O−H)−	3-Hydroxyisovaleric acid	AXFYFNCPONWUHW-UHFFFAOYSA-N
M172T334	172.0963852	334.071	(M−H)−	Acetyl-DL-Leucine	WXNXCEHXYPACJF-UHFFFAOYSA-N
M346T406	346.0522292	405.907	(M−H)−	Adenosine 3′-monophosphate	HAWIDQLWSQBRQS-MCDZGGTQSA-N
M346T431	346.0526705	431.025	(M−H)−	Adenosine monophosphate (AMP)	UDMBCSSLTHHNCD-KQYNXXCUSA-N
M353T396	353.0851513	395.847	(M−H)−	Chlorogenic acid	CWVRJTMFETXNAD-JUHZACGLSA-N
M147T463	147.0290525	462.523	(M−H)−	Citramalic acid	XFTRTWQBIOMVPK-UHFFFAOYSA-N
M161T305	161.0449936	305.073	(M−H_2_O−H)−	D-Tagatose	LKDRXBCSQODPBY-OEXCPVAWSA-N
M89T94	89.02443066	93.627	(M−H)−	DL-lactate	JVTAAEKCZFNVCJ-UHFFFAOYSA-M
M199T48_2	199.1701096	48.22	(M−H)−	Dodecanoic acid	POULHZVOKOAJMA-UHFFFAOYSA-N
M275T399	275.0878806	399.187	(M−H)−	gamma-L-Glutamyl-L-glutamic acid	OWQDWQKWSLFFFR-WDSKDSINSA-N
M601T32_3	601.3605355	31.541	(M−H)−	Garcinol	DTTONLKLWRTCAB-BZSUNBQASA-N
M267T217	267.0728763	217.239	(M−H)−	Inosine	UGQMRVRMYYASKQ-KQYNXXCUSA-N
M209T207	209.0654233	207.029	(M+CH_3_COO)−	L-Arabinose	SRBFZHDQGSBBOR-HWQSCIPKSA-N
M174T395	174.0399605	395.197	(M−H)−	N-Acetyl-L-aspartic acid	OTCCIMWXFLJLIA-BYPYZUCNSA-N
M662T436	662.0984624	436.395	(M−H)−	Nicotinamide adenine dinucleotide (NAD)	BAWFJGJZGIEFAR-NNYOXOHSSA-N
M339T123	339.1951453	123.415	(M−H)−	Norethindrone acetate	IMONTRJLAWHYGT-ZCPXKWAGSA-N
M255T44	255.2323813	43.66	(M−H)−	Palmitic acid	IPCSVZSSVZVIGE-UHFFFAOYSA-N
M163T56	163.0389977	55.969	(M−H)−	Phenylpyruvate	BTNMPGBKDVTSJY-UHFFFAOYSA-N
M73T393_2	73.02972583	392.588	(M−H)−	Propionic acid	XBDQKXXYIPTUBI-UHFFFAOYSA-N
M173T143	173.0411872	143.313	(M−H)−	Shikimic acid	JXOHGGNKMLTUBP-HSUXUTPPSA-N
M341T675	341.1065538	675.4475	(M−H)−	Trehalose	HDTRYLNUVZCQOY-LIZSDCNHSA-N
M303T163	303.0798431	162.842	(M+CH_3_COO)−	Uridine	DRTQHJPVMGBUCF-XVFCMESISA-N
M167T48	167.0346289	48.22	(M−H)−	Vanillic acid	WKOLLVMJNQIZCI-UHFFFAOYSA-N
M468T195	468.3062751	195.351	(M+H)+	1-Myristoyl-sn-glycero-3-phosphocholine	VXUOFDJKYGDUJI-OAQYLSRUSA-N
M210T153	210.0745414	152.704	(M+CH_3_CN+Na)+	2,2-Dimethyl Succinic acid	BCUIGUVXRMEZDV-UHFFFAOYSA-N
M282T133	282.1186045	132.585	(M+H)+	2′-O-Methyladenosine	FPUGCISOLXNPPC-UHFFFAOYSA-N
M207T128	207.0634386	127.975	(M+H−H_2_O)+	3,5-Dimethoxy-4-hydroxycinnamic acid	GFDHKVZSFFTEST-ONEGZZNKSA-N
M146T257_2	146.0584312	256.988	(M+H−2H_2_O)+	DL-O-tyrosine	WRFPVMFCRNYQNR-UHFFFAOYSA-N
M261T440	261.0350603	439.926	(M−2H+3Na)+	D-Pinitol	DSCFFEYYQKSRSV-FEPQRWDDSA-N
M203T258	203.1384339	258.347	(M+H)+	Ile-Ala	RCFDOSNHHZGBOY-ACZMJKKPSA-N
M287T129	287.0531181	129.315	(M+H)+	Kaempferol	IYRMWMYZSQPJKC-UHFFFAOYSA-N
M205T256	205.0966437	256.257	(M+H)+	L-Tryptophan	QIVBCDIJIAJPQS-VIFPVBQESA-N
M99T181	99.0424654	181.012	(M+H−H_2_O)+	Methyl acetoacetate	WRQNANDWMGAFTP-UHFFFAOYSA-N
M303T230	303.0486481	229.629	(M+H)+	Morin	YXOLAZRVSSWPPT-UHFFFAOYSA-N
M222T276_2	222.0955499	276.476	(M+H)+	N-Acetyl-D-glucosamine	OVRNDRQMDRJTHS-RTRLPJTCSA-N
M567T232	567.1316732	231.649	(2M+Na)+	Naringenin	FTVWIRXFELQLPI-UHFFFAOYSA-N
M152T219	152.0690778	219.09	(M+H)+	N-Methylanthranilic acid	WVMBPWMAQDVZCM-UHFFFAOYSA-N
M122T641	122.0952721	641.345	(M+H)+	N,N-Dimethylaniline	JLTDJTHDQAWBAV-UHFFFAOYSA-N
M206T73	206.1368936	72.819	(M+H)+	Pantothenol	SNPLKNRPJHDVJA-ZETCQYMHSA-N
M297T253	297.1195268	252.778	(M+H)+	Phe-Met	PYOHODCEOHCZBM-RYUDHWBXSA-N
M131T272_2	131.0519407	272.316	(M+H−2H_2_O)+	Phenyllactic acid	NWCHELUCVWSRRS-UHFFFAOYSA-N
M248T135	248.121078	135.195	(M+NH_4_)+	Pro-Asp	GLEOIKLQBZNKJZ-WDSKDSINSA-N
M300T149	300.2883209	148.614	(M+H)+	Sphingosine	WWUZIQQURGPMPG-KRWOKUGFSA-N
M267T209	267.1304837	209.1855	(M+H)+	Thr-Phe	IQHUITKNHOKGFC-MIMYLULJSA-N
M351T275	351.1310253	275.056	(M+Na)+	Tyr-Phe	CGWAPUBOXJWXMS-UHFFFAOYSA-N
M304T196	304.1647243	196.071	(M+H)+	Val-Trp	LZDNBBYBDGBADK-KBPBESRZSA-N
M469T159	469.3363531	158.874	(M−H)−	11-keto-.beta.-Boswellic acid	YIMHGPSYDOGBPI-IQQSWPBXSA-N
M89T83	89.02414079	82.519	(M−H)−	3-Hydroxypropionic acid (beta-lactic acid)	ALRHLSYJTWAHJZ-UHFFFAOYSA-N
M175T249	175.0239258	249.299	(M−H_2_O−H)−	D-Glucuronate	AEMOLEFTQBMNLQ-AQKNRBDQSA-N
M339T500	338.9854391	499.873	(2M−H)−	Dihydroxyacetone phosphate	GNGACRATGGDKBX-UHFFFAOYSA-N
M165T123	165.0542302	123.217	(M−H)−	DL-3-Phenyllactic acid	VOXXWSYKYCBWHO-UHFFFAOYSA-N
M259T317	259.0214492	316.684	(M−H)−	Fructose 1-phosphate	RHKKZBWRNHGJEZ-ARQDHWQXSA-N
M269T45_2	269.2471566	45.372	(M−H)−	Heptadecanoic acid	KEMQGTRYUADPNZ-UHFFFAOYSA-N
M204T249_1	204.085778	248.649	(M+CH_3_COO)−	Isobutyrylglycine	DCICDMMXFIELDF-UHFFFAOYSA-N
M160T410	160.0606898	410.448	(M−H)−	L-2-Aminoadipic acid	OYIFNHCXNCRBQI-BYPYZUCNSA-N
M115T426	115.0039537	425.657	(M−H)−	Maleic acid	VZCYOOQTPOCHFL-UPHRSURJSA-N
M503T416	503.158231	415.578	(M−H)−	Melezitose	QWIZNVHXZXRPDR-WSCXOGSTSA-N
M129T454	129.0188738	453.615	(M−H)−	Mesaconic acid	HNEGQIOMVPPMNR-NSCUHMNNSA-N
M433T167	433.111225	167.194	(M−H)−	Naringenin-7-O-Glucoside	DLIKSSGEMUFQOK-SFTVRKLSSA-N
M455T39	455.3528301	38.762	(M−H)−	Oleanolic acid	MIJYXULNPSFWEK-GTOFXWBISA-N
M349T8	349.2346927	7.5065	(M−H)−	Tetrahydrocorticosterone	RHQQHZQUAMFINJ-DTDWNVJFSA-N

**Table 2:** Detailed ADME information of the compound.

Name	Inchikey	MW	AlogP	Hdon	Hacc	OB (%)	Caco-2	BBB	DL	FASA	TPSA	RBN	HL
L-Phenylalanine	COLNVLDHVKWLRT-QMMMGPOBSA-N	165.21	0.96	3	3	41.62	0.36	0.22	0.04	63.32	0	3	4.62
L-Glutamate	WHUUTDBJXJRKMK-VKHMYHEASA-N	147.15	−0.92	4	5	6.66	−1.05	−1.97	0.02	100.62	0	4	0
L-Arginine	ODKSFYDXXFIFQN-BYPYZUCNSA-N	174.24	−1.11	7	6	47.64	−0.49	−1.04	0.03	125.22	0	6	0.85
L-Lysine	KDXKERNSBIXSRK-YFKPBYRVSA-N	146.22	−0.68	5	4	29.33	−0.66	−1.44	0.02	89.34	0	5	0
Uridine	DRTQHJPVMGBUCF-XVFCMESISA-N	244.23	−2.45	4	8	10.49	−1.14	−1.61	0.11	124.78	0	2	0
L-Aspartate	CKLJMWTZIZZHCS-REOHCLBHSA-N	133.12	−1.24	4	5	79.74	−1.02	−1.53	0.02	100.62	0	3	11.38
Palmitic acid	IPCSVZSSVZVIGE-UHFFFAOYSA-N	256.48	6.37	1	2	19.3	1.09	1	0.1	37.3	0	14	0
L-Histidine	HNDVDQJCIGZPNO-YFKPBYRVSA-N	155.18	−1.01	4	4	53.18	−0.25	−0.4	0.03	92	0	3	−5.72
Quercetin	REFJWTPEDVJJIY-UHFFFAOYSA-N	302.25	1.5	5	7	46.43	0.05	−0.77	0.28	131.36	0.38	1	14.4
Vanillic acid	WKOLLVMJNQIZCI-UHFFFAOYSA-N	168.16	1.15	2	4	35.47	0.43	0.09	0.04	66.76	0.34	2	11.62
Linoleic acid	OYHQOLUKZRVURQ-HZJYTTRNSA-N	280.5	6.39	1	2	41.9	1.16	0.9	0.14	37.3	0.25	14	7.5
Oleanolic acid	MIJYXULNPSFWEK-GTOFXWBISA-N	456.78	6.42	2	3	29.02	0.59	0.07	0.76	57.53	0.25	1	0
Dodecanoic acid	POULHZVOKOAJMA-UHFFFAOYSA-N	200.36	4.54	1	2	23.59	1.02	1.1	0.04	37.3	0	10	0
trans-Ferulic acid	KSEBMYQBYZTDHS-HWKANZROSA-N	194.2	1.62	2	4	39.56	0.47	−0.03	0.06	66.76	0.34	3	2.38
4-Aminobutyric acid	BTCSSZJGUNDROE-UHFFFAOYSA-N	103.14	−0.62	3	3	24.09	−0.26	−0.57	0.01	63.32	0	3	0
Kaempferol	IYRMWMYZSQPJKC-UHFFFAOYSA-N	286.25	1.77	4	6	41.88	0.26	−0.55	0.24	111.13	0	1	14.74
L-Asparagine	DCXYFEDJOCDNAF-REOHCLBHSA-N	132.14	−1.85	5	5	83.96	−0.88	−1.15	0.02	106.41	0	3	11.59
alpha-Linolenic acid	DTOSIQBPPRVQHS-PDBXOOCHSA-N	278.48	5.95	1	2	45.01	1.21	0.84	0.15	37.3	0	13	5.54
1,3,5-Benzenetriol	QCDYQQDYXPDABM-UHFFFAOYSA-N	126.12	1.03	3	3	24.34	0.33	−0.06	0.02	60.69	0	0	0
(+)-Catechin	PFTAWBLQPZVEMU-DZGCQCFKSA-N	290.29	1.92	5	6	54.83	−0.03	−0.73	0.24	110.38	0	1	0.61
Trehalose	HDTRYLNUVZCQOY-LIZSDCNHSA-N	342.34	−4.26	8	11	2.32	−3.08	−7	0.24	189.53	0.23	4	0
Astragalin	JPUKWEQWGBDDQB-QSOFNFLRSA-N	448.41	−0.32	7	11	14.03	−1.34	−1.97	0.74	190.28	0.34	4	0
Erythritol	UNXHWFMMPAWVPI-ZXZARUISSA-N	122.14	−1.92	4	4	59.62	−1.3	−3.32	0.01	80.92	0.21	3	11.25
Quercitrin	OXGUCUVFOIWWQJ-HQBVPOQASA-N	448.41	0.3	7	11	4.04	−1.04	−1.94	0.74	190.28	0.33	3	0
Stachyose	UQZIYBXSHAGNOE-XNSRJBNMSA-N	666.66	−7.8	14	21	3.25	−5.54	−12.76	0.59	347.83	0.21	11	0
Morin	YXOLAZRVSSWPPT-UHFFFAOYSA-N	302.25	1.5	5	7	46.23	0	−0.77	0.27	131.36	0.41	1	15.51
4-Hydroxycinnamic acid	NGSWKAQJJWESNS-ZZXKWVIFSA-N	164.17	1.64	2	3	43.29	0.46	0.13	0.04	57.53	0.45	2	4.43
Raffinose	MUPFEKGTMRGPLJ-ZQSKZDJDSA-N	504.5	−6.06	11	16	11.79	−3.91	−8.77	0.66	268.68	0.21	8	0
Sucrose	CZMRCDWAGMRECN-UGDNZRGBSA-N	342.34	−4.31	8	11	7.17	−2.89	−6.67	0.23	189.53	0.2	5	0
Nicotinamide	DFPAKSUCGFBDDF-UHFFFAOYSA-N	122.14	−0.32	2	3	71.13	0.44	0.2	0.02	55.98	0.33	1	11.89
Stearic acid	QIQXTHQIDYTFRH-UHFFFAOYSA-N	284.54	7.28	1	2	17.83	1.15	1.22	0.14	37.3	0.19	16	0
Erucamide	UAUDZVJPLUQNMU-KTKRTIGZSA-N	337.66	8.06	2	2	27.85	1.27	0.75	0.26	43.09	0.18	19	0
4-Guanidinobutyric acid	TUHVEAJXIMEOSA-UHFFFAOYSA-N	145.19	−0.59	5	5	47.74	0.07	−0.28	0.02	99.2	0.27	5	−3.52
L-Malic acid	BJEPYKJPYRNKOW-REOHCLBHSA-N	134.1	−0.95	3	5	59.62	−0.87	−1.4	0.02	94.83	0.41	3	11.4
Tyramine	DZGWFCGJZKJUFP-UHFFFAOYSA-N	137.2	0.99	3	2	45.11	0.74	0.52	0.02	46.25	0.32	2	−2.52
Heptadecanoic acid	KEMQGTRYUADPNZ-UHFFFAOYSA-N	270.51	6.82	1	2	18.51	1.12	0.95	0.12	37.3	0.21	15	0
Phloridzin	IOUVKUPGCMBWBT-QNDFHXLGSA-N	436.45	0.75	7	10	2.88	−1.23	−2.02	0.6	177.14	0.34	7	0
2-Hydroxyadenine	DRAVOWXCEBXPTN-UHFFFAOYSA-N	151.15	−0.19	4	5	68.19	−0.55	−1.01	0.04	100.71	0.28	0	13.56
alpha-D-Glucose	WQZGKKKJIJFFOK-DVKNGEFBSA-N	180.18	−2.51	5	6	50.38	−1.93	−4.47	0.04	110.38	0.27	1	11.12
cis-9-Palmitoleic acid	SECPZKHBENQXJG-FPLPWBNLSA-N	254.46	5.92	1	2	35.78	1.18	0.88	0.1	37.3	0.24	13	5.29
Indole	SIKJAQJRHWYJAI-UHFFFAOYSA-N	117.16	2.12	1	0	34.38	1.81	2.07	0.03	15.79	0.2	0	5.56
L-Tryptophan	QIVBCDIJIAJPQS-VIFPVBQESA-N	204.25	1.25	4	3	75.93	0.26	−0.17	0.08	79.11	0.27	3	−2.49
Adenosine	OIRDTQYFTABQOQ-KQYNXXCUSA-N	267.28	−2.02	5	8	15.98	−1.56	−2.22	0.18	139.54	0.23	2	0
Adenine	GFFGJBXGBJISGV-UHFFFAOYSA-N	135.15	−0.58	3	4	62.81	−0.3	−0.63	0.03	80.48	0	0	13.33
Maleic acid	VZCYOOQTPOCHFL-UPHRSURJSA-N	116.08	−0.01	2	4	65.06	−0.46	−0.73	0.01	74.6	0.35	2	12.07
Salicylic acid	YGSDEFSMJLZEOE-UHFFFAOYSA-N	138.13	1.17	2	3	32.13	0.63	0.63	0.03	57.53	0.43	1	12
Chlorogenic acid	CWVRJTMFETXNAD-JUHZACGLSA-N	354.34	−0.42	6	9	11.93	−1.03	−1.71	0.33	164.75	0.37	5	0
Lanosterol	CAHGCLMLTWQZNJ-BQNIITSRSA-N	426.8	8.12	1	1	42.12	1.52	1.18	0.75	20.23	0.23	4	5.84
Myricetin	IKMDFBPHZNJCSN-UHFFFAOYSA-N	318.25	1.24	6	8	13.75	−0.15	−1.01	0.31	151.59	0.4	1	0
Anthranilic acid (vitamin L1)	RWZYAGGXGHYGMB-UHFFFAOYSA-N	137.15	0.69	3	3	60.35	0.25	0.15	0.03	63.32	0.41	1	11.9
L-Pyroglutamic acid	ODHCTXKNWHHXJC-VKHMYHEASA-N	129.13	−0.67	2	4	96.25	−0.2	−0.26	0.02	66.4	0.34	1	11.35
Quercetin 3′-methyl ether	WEPBGSIAWZTEJR-UHFFFAOYSA-N	316.28	1.57	4	7	10.1	0.2	−0.73	0.3	120.36	0.36	2	0
4-Hydroxybutanoic acid lactone	YEJRWHAVMIAJKC-UHFFFAOYSA-N	86.1	0.28	0	2	76.91	1.03	1.43	0.01	26.3	0.27	0	11.45
All cis-(6,9,12)-Linolenic acid	VZCCETWTMQHEPK-QNEBEIHSSA-N	278.48	5.95	1	2	45.01	1.2	0.7	0.15	37.3	0.27	13	5.97
Isovaleric acid	GWYFCOCPABKNJV-UHFFFAOYSA-N	102.15	1.15	1	2	62.17	0.82	1.09	0.01	37.3	0.29	2	11.36
D-Threitol	UNXHWFMMPAWVPI-QWWZWVQMSA-N	122.14	−1.92	4	4	46.41	−1.13	−2.97	0.01	80.92	0.19	3	11.3
L-Pipecolic acid	HXEACLLIILLPRG-YFKPBYRVSA-N	129.18	0.4	2	3	66.14	0.32	0.38	0.02	49.33	0.28	1	11.08
L-Threonine	AYFVYJQAPQTCCC-GBXIJSLDSA-N	119.14	−1.11	4	4	73.52	−0.87	−2.56	0.01	83.55	0.3	2	11.42
Hyperoside	OVSQVDMCBVZWGM-DTGCRPNFSA-N	464.41	−0.59	8	12	6.94	−1.42	−2.08	0.77	210.51	0	4	0
Dihydroxyacetone	RXKJFZQQPQGTFL-UHFFFAOYSA-N	90.09	−1.16	2	3	58.6	−0.56	−1.04	0.01	57.53	0.29	2	11.79
Propionic acid	XBDQKXXYIPTUBI-UHFFFAOYSA-N	74.09	0.44	1	2	93.06	0.6	0.89	0	37.3	0.31	1	11.81
trans-3-Coumaric acid	KKSDGJDHHZEWEP-SNAWJCMRSA-N	164.17	1.64	2	3	49.54	0.45	0.12	0.04	57.53	0.45	2	1.99
(+-)-Taxifolin	CXQWRCVTCMQVQX-LSDHHAIUSA-N	304.27	1.49	5	7	57.84	−0.23	−0.8	0.27	127.45	0.39	1	14.41
Phenylacetic acid	WLJVXDMOQOGPHL-UHFFFAOYSA-N	136.16	1.47	1	2	72.35	0.84	0.97	0.02	37.3	0.43	2	−2.03
Dihydrouracil	OIVLITBTBDPEFK-UHFFFAOYSA-N	114.12	−0.91	2	4	67.9	0.07	0.07	0.02	58.2	0.3	0	11.41
cis-Aconitate	GTZCVFVGUGFEME-IWQZZHSRSA-N	174.12	−0.41	3	6	11.12	−0.82	−1.23	0.04	111.9	0.42	4	0
Shikimate	JXOHGGNKMLTUBP-HSUXUTPPSA-N	174.17	−1.18	4	5	46.24	−1.16	−1.56	0.04	97.99	0.32	1	11.18
Diosmin	GZSOSUNBTXMUFQ-YFAPSIMESA-N	608.6	−0.44	8	15	12.7	−1.93	−2.7	0.66	238.2	0.29	7	0
(+)-Abscisic acid	JLIDBLDQVAYHNE-YKALOCIXSA-N	264.35	2	2	4	63.67	0.13	−0.2	0.13	74.6	0.35	3	5.51
Dopamine	VYFYYTLLBUKUHU-UHFFFAOYSA-N	153.2	0.72	4	3	74.4	0.3	−0.21	0.03	66.48	0.34	2	1.84
L-Leucine	ROHFNLRQFUQHCH-YFKPBYRVSA-N	131.2	0.63	3	3	72.92	−0.05	−0.37	0.01	63.32	0.29	3	11.41
L-Methionine	FFEARJCKVFRZRR-BYPYZUCNSA-N	149.24	−0.27	3	3	70.87	0.06	−0.17	0.01	88.62	0.37	4	11.69
Abscisic acid (cis, trans)	JLIDBLDQVAYHNE-QHFMCZIYSA-N	264.35	2	2	4	31.79	0.06	−0.26	0.13	74.6	0.34	3	5.61
Maslinic Acid	MDZKJHQSJHYOHJ-LLICELPBSA-N	472.78	5.46	3	4	15.54	0.1	−0.55	0.74	77.76	0.25	1	0
N-Methylanthranilic Acid	WVMBPWMAQDVZCM-UHFFFAOYSA-N	151.18	1.24	2	3	52.98	0.98	1.04	0.03	49.33	0.4	2	31.05
Linustatin	FERSMFQBWVBKQK-CXTTVELOSA-N	409.44	−3.32	7	12	2.54	−2.15	−2.86	0.41	202.32	0.28	6	0
3-Hydroxy-4-methoxycinnamic acid	QURCVMIEKCOAJU-HWKANZROSA-N	194.2	1.62	2	4	50.83	0.49	0.01	0.06	66.76	0	3	2.45
Uvaol	XUARCIYIVXVTAE-ZAPOICBTSA-N	442.8	6.26	2	2	17.13	0.73	−0.01	0.76	40.46	0.23	1	0
Homogentisic acid	IGMNYECMUMZDDF-UHFFFAOYSA-N	168.16	0.93	3	4	92.44	0.24	0	0.04	77.76	0.41	2	4.79
Epigallocatechin gallate	WMBWREPUVVBILR-WIYYLYMNSA-N	458.4	2.89	8	11	55.09	−0.57	−1.7	0.77	197.37	0.37	4	1.7
D-Galactarate	DSLZVSRJTYRBFB-DUHBMQHGSA-N	210.16	−2.52	6	8	15.96	−2.43	−5.33	0.06	155.52	0.4	5	0
Guanosine	NYHBQMYGNKIUIF-UUOKFMHZSA-N	283.28	−2.41	6	9	20.9	−1.22	−1.91	0.21	159.51	0.27	2	0
Mesaconic acid	HNEGQIOMVPPMNR-NSCUHMNNSA-N	130.11	0.44	2	4	69.77	−0.3	−0.53	0.02	74.6	0.38	2	11.8
Procyanidin B2	XFZJEEAOWLFHDH-NFJBMHMQSA-N	578.56	3.36	10	12	3.01	−1.14	−2.02	0.66	220.76	0.32	3	0
Procyanidin C1	MOJZMWJRUKIQGL-XILRTYJMSA-N	866.83	4.8	15	18	18.98	−1.99	−3.86	0.1	331.14	0.34	5	0
1,2-Benzenedicarboxylic acid	XNGIFLGASWRNHJ-UHFFFAOYSA-N	166.14	1.04	2	4	17.74	−0.05	−0.23	0.04	74.6	0.46	2	0
Inosine	UGQMRVRMYYASKQ-KQYNXXCUSA-N	268.26	−2.22	4	8	10.35	−1.26	−1.68	0.18	133.49	0.26	2	0
Naringenin-7-O-Glucoside	DLIKSSGEMUFQOK-SFTVRKLSSA-N	434.43	0.39	6	10	9.33	−1.23	−2.02	0.74	166.14	0.36	4	0
3-Dehydroshikimic acid	SLWWJZMPHJJOPH-PHDIDXHHSA-N	172.15	−0.93	3	5	46.09	−1.25	−2.06	0.04	94.83	0.38	1	11.52
Dehydroascorbic acid (Oxidized vitamin C)	SBJKKFFYIZUCET-JLAZNSOCSA-N	174.12	−1.63	2	6	65.67	−1.12	−1.39	0.04	100.9	0.49	2	11.48
Cytosine	OPTASPLRGRRNAP-UHFFFAOYSA-N	111.12	−0.99	3	4	50.04	0.31	0.33	0.02	71.77	0.32	0	11.83
D-Aspartic acid	CKLJMWTZIZZHCS-UWTATZPHSA-N	133.12	−1.24	4	5	70.57	−0.86	−1.14	0.02	100.62	0.4	3	11.4
D-Proline	ONIBWKKTOPOVIA-SCSAIBSYSA-N	115.15	−0.06	2	3	86.46	0.28	0.44	0.01	49.33	0.32	1	11.15
Sphingosine	WWUZIQQURGPMPG-KRWOKUGFSA-N	299.56	4.83	4	3	17.5	0.36	−0.29	0.16	66.48	0.16	15	0
trans-Vaccenic acid	UWHZIFQPPBDJPM-BQYQJAHWSA-N	282.52	6.84	1	2	33.13	1.17	0.95	0.14	37.3	0.23	15	5.44
Pyridoxine	LXNHXLLTXMVWPM-UHFFFAOYSA-N	169.2	−0.51	3	4	61.54	−0.16	−0.81	0.04	73.58	0.21	2	11.41
Phenylpyruvate	BTNMPGBKDVTSJY-UHFFFAOYSA-N	164.17	1.21	1	3	32.72	0.39	0.46	0.04	54.37	0.45	3	5.07
2,3-Dihydroxybenzoic acid	GLDQAMYCGOIJDV-UHFFFAOYSA-N	154.13	0.9	3	4	28.55	0.31	0.19	0.04	77.76	0.42	1	0
Cyclohexylamine	PAFZNILMFXTMIY-UHFFFAOYSA-N	99.2	1.21	2	1	86.34	1.06	1.14	0.01	26.02	0.17	0	10.86
D-Pipecolinic acid	HXEACLLIILLPRG-RXMQYKEDSA-N	129.18	0.4	2	3	59.88	0.41	0.51	0.02	49.33	0.27	1	11.03
Betaine	KWIUHFFTVRNATP-UHFFFAOYSA-N	341.29	2.9	1	7	24.8	0.36	−0.35	0.55	110.81	0.05	3	0
Pyruvaldehyde	AIJULSRZWUXGPQ-UHFFFAOYSA-N	NA	NA	NA	NA	NA	NA	NA	NA	NA	NA	NA	NA
N6,N6,N6-Trimethyl-L-lysine	MXNRLFUSFKVQSK-QMMMGPOBSA-N	NA	NA	NA	NA	NA	NA	NA	NA	NA	NA	NA	NA
Ribitol	HEBKCHPVOIAQTA-NGQZWQHPSA-N	NA	NA	NA	NA	NA	NA	NA	NA	NA	NA	NA	NA
myo-Inositol	CDAISMWEOUEBRE-UHFFFAOYSA-N	NA	NA	NA	NA	NA	NA	NA	NA	NA	NA	NA	NA
Glutathione	RWSXRVCMGQZWBV-WDSKDSINSA-N	NA	NA	NA	NA	NA	NA	NA	NA	NA	NA	NA	NA
Hydroxyproline	PMMYEEVYMWASQN-DMTCNVIQSA-N	NA	NA	NA	NA	NA	NA	NA	NA	NA	NA	NA	NA

**Table 3:** ADME information of the compound after screening.

Name	Inchikey	MW	AlogP	Hdon	Hacc	OB (%)	Caco-2	BBB	DL	FASA	TPSA	RBN	HL
Quercetin	REFJWTPEDVJJIY-UHFFFAOYSA-N	302.25	1.5	5	7	46.43	0.05	−0.77	0.28	131.36	0.38	1	14.4
Oleanolic acid	MIJYXULNPSFWEK-GTOFXWBISA-N	456.78	6.42	2	3	29.02	0.59	0.07	0.76	57.53	0.25	1	0
Kaempferol	IYRMWMYZSQPJKC-UHFFFAOYSA-N	286.25	1.77	4	6	41.88	0.26	−0.55	0.24	111.13	0	1	14.74
(+)-Catechin	PFTAWBLQPZVEMU-DZGCQCFKSA-N	290.29	1.92	5	6	54.83	−0.03	−0.73	0.24	110.38	0	1	0.61
Morin	YXOLAZRVSSWPPT-UHFFFAOYSA-N	302.25	1.5	5	7	46.23	0	−0.77	0.27	131.36	0.41	1	15.51
Erucamide	UAUDZVJPLUQNMU-KTKRTIGZSA-N	337.66	8.06	2	2	27.85	1.27	0.75	0.26	43.09	0.18	19	0
Lanosterol	CAHGCLMLTWQZNJ-BQNIITSRSA-N	426.8	8.12	1	1	42.12	1.52	1.18	0.75	20.23	0.23	4	5.84
(±)-Taxifolin	CXQWRCVTCMQVQX-LSDHHAIUSA-N	304.27	1.49	5	7	57.84	−0.23	−0.8	0.27	127.45	0.39	1	14.41
Epigallocatechin gallate	WMBWREPUVVBILR-WIYYLYMNSA-N	458.4	2.89	8	11	55.09	−0.57	−1.7	0.77	197.37	0.37	4	1.7
Guanosine	NYHBQMYGNKIUIF-UUOKFMHZSA-N	283.28	−2.41	6	9	20.9	−1.22	−1.91	0.21	159.51	0.27	2	0
Betaine	KWIUHFFTVRNATP-UHFFFAOYSA-N	341.29	2.9	1	7	24.8	0.36	−0.35	0.55	110.81	0.05	3	0

**Table 4:** Biological function enrichment results.

Category	Term	Count	*P* value	FDR
GOTERM_BP_DIRECT	GO:0038128∼ERBB2 signaling pathway	13	1.28*E* − 22	9.55*E* − 20
KEGG_PATHWAY	hsa04012:ErbB signaling pathway	17	1.27*E* − 20	1.01*E* − 18
GOTERM_BP_DIRECT	GO:0007173∼epidermal growth factor receptor signaling pathway	12	3.15*E* − 18	1.17*E* − 15
KEGG_PATHWAY	hsa05211:renal cell carcinoma	14	3.40*E* − 17	1.34*E* − 15
KEGG_PATHWAY	hsa05200:pathways in cancer	22	7.81*E* − 16	2.06*E* − 14
KEGG_PATHWAY	hsa05205:proteoglycans in cancer	17	1.23*E* − 14	2.43*E* − 13
GOTERM_BP_DIRECT	GO:0048015∼phosphatidylinositol-mediated signaling	11	2.78*E* − 13	6.89*E* − 11
GOTERM_CC_DIRECT	GO:0005829∼cytosol	32	7.05*E* − 13	7.90*E* − 11
GOTERM_BP_DIRECT	GO:0014066∼regulation of phosphatidylinositol 3-kinase signaling	10	7.39*E* − 13	1.38*E* − 10
GOTERM_MF_DIRECT	GO:0005515∼protein binding	47	1.96*E* − 12	2.97*E* − 10
KEGG_PATHWAY	hsa04722:neurotrophin signaling pathway	13	3.24*E* − 12	5.11*E* − 11
GOTERM_MF_DIRECT	GO:0046934∼phosphatidylinositol-4,5-bisphosphate 3-kinase activity	9	5.83*E* − 12	4.43*E* − 10
KEGG_PATHWAY	hsa05220:chronic myeloid leukemia	11	9.33*E* − 12	1.23*E* − 10
KEGG_PATHWAY	hsa05223:nonsmall cell lung cancer	10	2.91*E* − 11	3.29*E* − 10
GOTERM_MF_DIRECT	GO:0004713∼protein tyrosine kinase activity	10	9.41*E* − 11	4.77*E* − 09
GOTERM_BP_DIRECT	GO:0046854∼phosphatidylinositol phosphorylation	9	1.87*E* − 10	2.79*E* − 08
GOTERM_BP_DIRECT	GO:0018108∼peptidyl-tyrosine phosphorylation	10	3.46*E* − 10	4.30*E* − 08
KEGG_PATHWAY	hsa04910:insulin signaling pathway	12	3.57*E* − 10	3.53*E* − 09
GOTERM_BP_DIRECT	GO:0042059∼negative regulation of epidermal growth factor receptor signaling pathway	7	6.31*E* − 10	6.72*E* − 08
KEGG_PATHWAY	hsa05100:bacterial invasion of epithelial cells	10	6.37*E* − 10	5.59*E* − 09
KEGG_PATHWAY	hsa04510:focal adhesion	13	1.90*E* − 09	1.50*E* − 08
KEGG_PATHWAY	hsa04066:HIF-1 signaling pathway	10	4.19*E* − 09	3.01*E* − 08
KEGG_PATHWAY	hsa04014:Ras signaling pathway	13	5.47*E* − 09	3.60*E* − 08
GOTERM_BP_DIRECT	GO:2000145∼regulation of cell motility	6	1.29*E* − 08	1.20*E* − 06
GOTERM_MF_DIRECT	GO:0019903∼protein phosphatase binding	7	2.02*E* − 08	6.58*E* − 07
KEGG_PATHWAY	hsa05213:endometrial cancer	8	2.05*E* − 08	1.24*E* − 07
GOTERM_MF_DIRECT	GO:0005154∼epidermal growth factor receptor binding	6	2.16*E* − 08	6.58*E* − 07
GOTERM_MF_DIRECT	GO:0005088∼Ras guanyl-nucleotide exchange factor activity	8	2.84*E* − 08	7.20*E* − 07
GOTERM_BP_DIRECT	GO:0043547∼positive regulation of GTPase activity	13	3.34*E* − 08	2.76*E* − 06
GOTERM_BP_DIRECT	GO:0000165∼MAPK cascade	10	3.88*E* − 08	2.89*E* − 06
KEGG_PATHWAY	hsa05215:prostate cancer	9	4.30*E* − 08	2.43*E* − 07
GOTERM_BP_DIRECT	GO:0007165∼signal transduction	17	4.78*E* − 08	3.24*E* − 06
GOTERM_BP_DIRECT	GO:0042060∼wound healing	7	8.88*E* − 08	5.51*E* − 06
KEGG_PATHWAY	hsa05230:central carbon metabolism in cancer	8	9.01*E* − 08	4.74*E* − 07
KEGG_PATHWAY	hsa05214:glioma	8	1.00*E* − 07	4.96*E* − 07
GOTERM_BP_DIRECT	GO:0007169∼transmembrane receptor protein tyrosine kinase signaling pathway	7	2.65*E* − 07	1.52*E* − 05
GOTERM_MF_DIRECT	GO:0017124∼SH3 domain binding	7	9.17*E* − 07	1.99*E* − 05
GOTERM_BP_DIRECT	GO:0071364∼cellular response to epidermal growth factor stimulus	5	2.08*E* − 06	1.11*E* − 04
GOTERM_BP_DIRECT	GO:0008286∼insulin receptor signaling pathway	6	2.50*E* − 06	1.24*E* − 04
GOTERM_BP_DIRECT	GO:0001525∼angiogenesis	8	2.65*E* − 06	1.24*E* − 04
GOTERM_BP_DIRECT	GO:0007175∼negative regulation of epidermal growth factor-activated receptor activity	4	3.34*E* − 06	1.46*E* − 04
GOTERM_MF_DIRECT	GO:0004714∼transmembrane receptor protein tyrosine kinase activity	5	3.63*E* − 06	6.90*E* − 05
GOTERM_MF_DIRECT	GO:0046982∼protein heterodimerization activity	10	4.54*E* − 06	7.67*E* − 05
GOTERM_MF_DIRECT	GO:0001784∼phosphotyrosine binding	4	5.67*E* − 06	8.62*E* − 05
KEGG_PATHWAY	hsa05206:MicroRNAs in cancer	11	5.95*E* − 06	2.76*E* − 05
GOTERM_BP_DIRECT	GO:0000186∼activation of MAPKK activity	5	8.06*E* − 06	3.34*E* − 04
GOTERM_MF_DIRECT	GO:0019901∼protein kinase binding	9	8.27*E* − 06	1.14*E* − 04
GOTERM_BP_DIRECT	GO:0000187∼activation of MAPK activity	6	1.19*E* − 05	4.65*E* − 04
KEGG_PATHWAY	hsa04062:chemokine signaling pathway	9	1.32*E* − 05	5.79*E* − 05
GOTERM_CC_DIRECT	GO:0045121∼membrane raft	7	1.42*E* − 05	7.93*E* − 04
GOTERM_MF_DIRECT	GO:0005070∼SH3/SH2 adaptor activity	5	1.74*E* − 05	2.21*E* − 04
GOTERM_BP_DIRECT	GO:0043065∼positive regulation of apoptotic process	8	1.85*E* − 05	6.88*E* − 04
GOTERM_BP_DIRECT	GO:0050900∼leukocyte migration	6	2.24*E* − 05	7.67*E* − 04
GOTERM_BP_DIRECT	GO:0001892∼embryonic placenta development	4	2.27*E* − 05	7.67*E* − 04
KEGG_PATHWAY	hsa04915:estrogen signaling pathway	7	2.63*E* − 05	1.09*E* − 04
KEGG_PATHWAY	hsa05231:choline metabolism in cancer	7	2.95*E* − 05	1.14*E* − 04
KEGG_PATHWAY	hsa04151:PI3K-Akt signaling pathway	11	3.08*E* − 05	1.14*E* − 04
KEGG_PATHWAY	hsa04015:Rap1 signaling pathway	9	3.19*E* − 05	1.14*E* − 04
GOTERM_MF_DIRECT	GO:0042802∼identical protein binding	11	3.24*E* − 05	3.79*E* − 04
KEGG_PATHWAY	hsa05212:pancreatic cancer	6	4.19*E* − 05	1.44*E* − 04
GOTERM_MF_DIRECT	GO:0046875∼ephrin receptor binding	4	5.03*E* − 05	5.46*E* − 04
KEGG_PATHWAY	hsa05218:melanoma	6	6.42*E* − 05	2.03*E* − 04
KEGG_PATHWAY	hsa04917:prolactin signaling pathway	6	6.42*E* − 05	2.03*E* − 04
GOTERM_BP_DIRECT	GO:0008283∼cell proliferation	8	6.55*E* − 05	0.002121606
GOTERM_CC_DIRECT	GO:0005886∼plasma membrane	24	6.61*E* − 05	0.002469141
GOTERM_BP_DIRECT	GO:0072656∼maintenance of protein location in mitochondrion	3	7.63*E* − 05	0.002367512
GOTERM_MF_DIRECT	GO:0005168∼neurotrophin TRKA receptor binding	3	1.13*E* − 04	0.001145089
GOTERM_CC_DIRECT	GO:0005737∼cytoplasm	27	1.13*E* − 04	0.003168298
GOTERM_BP_DIRECT	GO:0043619∼regulation of transcription from RNA polymerase II promoter in response to oxidative stress	3	1.14*E* − 04	0.003272239
GOTERM_BP_DIRECT	GO:0072655∼establishment of protein localization to mitochondrion	3	1.14*E* − 04	0.003272239
GOTERM_BP_DIRECT	GO:0071902∼positive regulation of protein serine/threonine kinase activity	4	1.26*E* − 04	0.003485245
KEGG_PATHWAY	hsa04068:FoxO signaling pathway	7	1.44*E* − 04	4.38*E* − 04
GOTERM_BP_DIRECT	GO:0007507∼heart development	6	1.54*E* − 04	0.004099115
GOTERM_MF_DIRECT	GO:0030235∼nitric-oxide synthase regulator activity	3	2.10*E* − 04	0.001949528
GOTERM_CC_DIRECT	GO:0005634∼nucleus	27	2.18*E* − 04	0.004872016
GOTERM_MF_DIRECT	GO:0030971∼receptor tyrosine kinase binding	4	2.31*E* − 04	0.001949528
GOTERM_MF_DIRECT	GO:0016303∼1-phosphatidylinositol-3-kinase activity	4	2.31*E* − 04	0.001949528
KEGG_PATHWAY	hsa04810:regulation of actin cytoskeleton	8	2.43*E* − 04	7.11*E* − 04
GOTERM_BP_DIRECT	GO:0043066∼negative regulation of apoptotic process	8	2.52*E* − 04	0.006477735
GOTERM_MF_DIRECT	GO:0036312∼phosphatidylinositol 3-kinase regulatory subunit binding	3	2.70*E* − 04	0.002158104
GOTERM_BP_DIRECT	GO:0045741∼positive regulation of epidermal growth factor-activated receptor activity	3	2.73*E* − 04	0.006769871
GOTERM_BP_DIRECT	GO:0045944∼positive regulation of transcription from RNA polymerase II promoter	11	3.16*E* − 04	0.007600594
KEGG_PATHWAY	hsa04660:T cell receptor signaling pathway	6	3.26*E* − 04	9.20*E* − 04
GOTERM_MF_DIRECT	GO:0004716∼receptor signaling protein tyrosine kinase activity	3	3.37*E* − 04	0.002558199
GOTERM_BP_DIRECT	GO:0036092∼phosphatidylinositol-3-phosphate biosynthetic process	4	3.46*E* − 04	0.008053746
GOTERM_BP_DIRECT	GO:0007266∼Rho protein signal transduction	4	3.67*E* − 04	0.008291918
GOTERM_MF_DIRECT	GO:0043560∼insulin receptor substrate binding	3	4.11*E* − 04	0.002972513
KEGG_PATHWAY	hsa04010:MAPK signaling pathway	8	7.50*E* − 04	0.002042501
KEGG_PATHWAY	hsa04650:natural killer cell mediated cytotoxicity	6	8.13*E* − 04	0.002141603
GOTERM_MF_DIRECT	GO:0005096∼GTPase activator activity	6	0.001034663	0.00714858
KEGG_PATHWAY	hsa05160:hepatitis C	6	0.001200247	0.003058693
KEGG_PATHWAY	hsa04973:carbohydrate digestion and absorption	4	0.002059125	0.005083466

## Data Availability

Data used to support this study are available on request from the corresponding author.

## Supplementary Materials

Supplementary Materials Supplementary schedule 1 mainly stores ADME data related to compounds screened in SD.
